# Outcomes of combined lung cancer resection and lung volume reduction surgery in adults with severe emphysema; a systematic review and meta-analysis

**DOI:** 10.3389/fonc.2026.1916431

**Published:** 2026-08-19

**Authors:** Ahmad Asqalan, Munir Adamu Bala, Ra’fat Tawalbeh, Ayush Jha, Beshoy Effat Elkomos, Mohammad Tayeh, Jameel Ismail Ahmad, Vasileios Kouritas

**Affiliations:** 1 Department of Thoracic Surgery, Norfolk and Norwich University Hospital, Norwich, United Kingdom; 2 General Surgery, Royal United Hospital, Bath, United Kingdom; 3 Faculty of Medicine, Jordan University of Science and Technology, Irbid, Jordan; 4 Cardiothoracic Surgery, Aminu Kano Teaching Hospital, Kano, Nigeria

**Keywords:** chronic obstructive pulmonary disease, emphysema, lobectomy, lung cancer, lung volume reduction surgery, meta-analysis, non-small-cell lung cancer, sublobar resection

## Abstract

**Background:**

Severe emphysema can preclude potentially curative lung cancer resection. In selected patients, a volume-reduction strategy may allow cancer resection while removing hyperinflated, poorly functioning parenchyma.

**Methods:**

We systematically reviewed adults with severe emphysema undergoing lung cancer resection combined with LVRS, LVRS-like resection, or lobar resection producing a volume-reduction effect. PubMed, Semantic Scholar, OpenAlex, Scopus, Web of Science Core Collection, CENTRAL, ClinicalTrials.gov, the WHO ICTRP, and reference lists were searched to 27 May 2026. Primary outcomes were perioperative mortality and major complications. Secondary outcomes included FEV1 change, exercise capacity, dyspnoea, quality of life, survival, and recurrence. Single-arm meta-analyses were performed when at least three comparable studies reported extractable data.

**Results:**

Ten unique datasets met the review criteria; seven contributed to at least one quantitative synthesis. Three deaths occurred among 113 participants (crude mortality 2.7%); the sparse-event random-effects logit model estimated 7.3% (95% CI 3.4–15.1). FEV1 improved by a pooled mean of +0.26 L (95% CI + 0.20 to +0.32; four studies, 74 patients). Reported 5-year overall survival ranged from 35% to 68% in the most informative cohorts, but survival and recurrence data were too heterogeneous for definitive oncologic conclusions.

**Conclusions:**

In highly selected patients with severe emphysema and resectable lung cancer, combined resection and LVRS appears feasible and may preserve or improve pulmonary function. Only 3/113 perioperative deaths (2.7%) were directly observed; the 7.3% pooled model estimate is not an observed risk and is unstable with only three deaths. The procedure should not be interpreted as low risk: it carries substantial air-leak morbidity, the certainty of evidence is very low. These findings support specialist multidisciplinary consideration, not routine use. Candidate selection should require concordance between curative oncologic resectability and a CT-defined, poorly perfused LVRS target, together with acceptable global reserve after optimisation.

**Systematic Review Registration:**

https://www.crd.york.ac.uk/PROSPERO, identifier CRD420261397321.

## Highlights

- Selected patients with early-stage lung cancer and severe emphysema may remain candidates for surgical resection despite limited predicted postoperative lung function.
- Combined lung cancer resection and lung volume reduction surgery was associated with a pooled FEV1 gain of 0.26 L in patients with favourable LVRS anatomy.
- Prolonged air leak occurred in approximately 38% of patients undergoing combined surgery.
- Patient selection should consider emphysema distribution, perfusion, resectability, pulmonary rehabilitation status, and the risk of prolonged air leak.
- These procedures should be considered in experienced thoracic surgical units following expert multidisciplinary team (MDT) assessment.

## Introduction

Lung cancer and emphysema frequently coexist in smokers, and severe emphysema complicates radiologic surveillance, operative-risk assessment, and postoperative recovery. For early-stage non-small-cell lung cancer (NSCLC), surgical resection remains the benchmark curative treatment, but severe physiological limitation often leads clinicians to deny lobectomy, restrict the extent of resection, or select non-operative local therapy instead (1–5).

The resurgence of lung volume reduction surgery (LVRS) in the 1990s created a different physiological premise. In carefully selected patients with hyperinflation and heterogeneous emphysematous destruction, removing the most diseased lung can improve elastic recoil, diaphragmatic mechanics, ventilation-perfusion matching, dyspnoea, exercise capacity, and FEV1 (1–3, 6–9). Early reports therefore proposed that lung cancer resection could be combined with LVRS when the tumour lay within, or could be resected alongside, a poorly perfused target area (1–3, 6–8).

This strategy has a clear physiological rationale but remains difficult to evaluate rigorously. Most reports are small, retrospective, and single-centre. The operations span three related but non-identical concepts: formal LVRS performed in addition to cancer resection, and LVRS-like sublobar resection that removes the tumour and target parenchyma together, and anatomical lobectomy of a poorly functioning target lobe that itself produces a lobar volume-reduction effect (2–4, 6–8, 10). Some cohorts include benign pulmonary nodules or mixed thoracic neoplasms rather than cancer-only populations, while others are superseded by later institutional reports or provide contextual rather than directly poolable evidence (5, 11–17). These features make uncritical pooling misleading unless overlap, endpoint compatibility, and surgical intent are handled explicitly.

We therefore performed a systematic review and meta-analysis of lung cancer resection combined with LVRS or a volume-reduction concept in adults with severe emphysema. We aimed to quantify perioperative mortality, prolonged air leak, and FEV1 change where data were sufficiently compatible, and to synthesize the broader functional and oncologic evidence narratively.

## Methods

### Protocol and reporting

The review question, eligibility criteria, outcomes, and synthesis rules were pre-specified and registered prospectively on PROSPERO (registration number: CRD420261397321). The manuscript was prepared in accordance with PRISMA 2020 (18). Risk-of-bias and certainty judgements were used to pre-specify sensitivity analyses and to avoid pooling clinically incompatible outcomes.

### Eligibility criteria

Eligible studies included adults aged at least 18 years with severe emphysema, COPD with severe emphysematous morphology, or LVRS candidacy, and radiologically or histologically confirmed lung cancer or suspected lung cancer where cancer-specific data were extractable. Eligible interventions were lung cancer resection performed simultaneously with formal LVRS, LVRS-like sublobar resection, or anatomical resection of a tumour-bearing emphysematous target lobe producing a volume-reduction effect. Comparators, when present, included tumour resection alone, standard lobectomy, LVRS-only cohorts, or risk strata defined by predicted postoperative FEV1. Case series with at least five patients, cohort studies, non-randomized studies, randomized studies, and conference abstracts with sufficient data were eligible. Only published or publicly citable outcome data were included. Case reports and reviews were excluded from synthesis.

Terminology was standardized as follows. ‘Combined resection–LVRS’ is the umbrella term; ‘formal LVRS’ denotes a separately described volume-reduction resection; ‘LVRS-like sublobar resection’ denotes a single sublobar specimen serving both oncologic and volume-reduction intent; ‘lobar volume-reduction effect’ denotes target-lobe anatomical resection without additional LVRS. ‘Lung volume reduction’ (LVR) describes the physiological concept and is not used as a fourth procedure category. Mixed pulmonary-nodule/neoplasm cohorts were eligible for nononcologic feasibility or functional synthesis only when the planned intervention applied uniformly; cancer-specific oncologic outcomes had to be extractable. Strict confirmed-malignancy sensitivity analyses excluded mixed cohorts whose safety or functional denominators could not be separated.

The primary outcomes were perioperative mortality, defined as 30-day, in-hospital, operative, or clearly reported early postoperative death, and major postoperative complications. Prolonged air leak was selected as the principal pooled morbidity endpoint because it was the most consistently reported complication. Secondary outcomes included FEV1 and FEV1% predicted, DLCO, residual volume, 6-minute walk distance, dyspnoea, quality of life, length of stay, recurrence, disease-free survival, and overall survival.

### Search strategy and study selection

We searched PubMed, Semantic Scholar, OpenAlex, Scopus, Web of Science Core Collection, CENTRAL, ClinicalTrials.gov, and the WHO International Clinical Trials Registry Platform from inception to 27 May 2026. Reference lists of included studies and related reviews were searched manually. Search concepts combined emphysema/COPD, LVRS or volume reduction, lung cancer/NSCLC, resection, lobectomy, wedge resection, and combined/concomitant/simultaneous procedures. No language restriction was applied; the Teschner 2003 paper (reference 13), published in German, was extracted by a German-speaking reviewer. No non-English records met eligibility criteria in the final included set.

**Figure 1:**
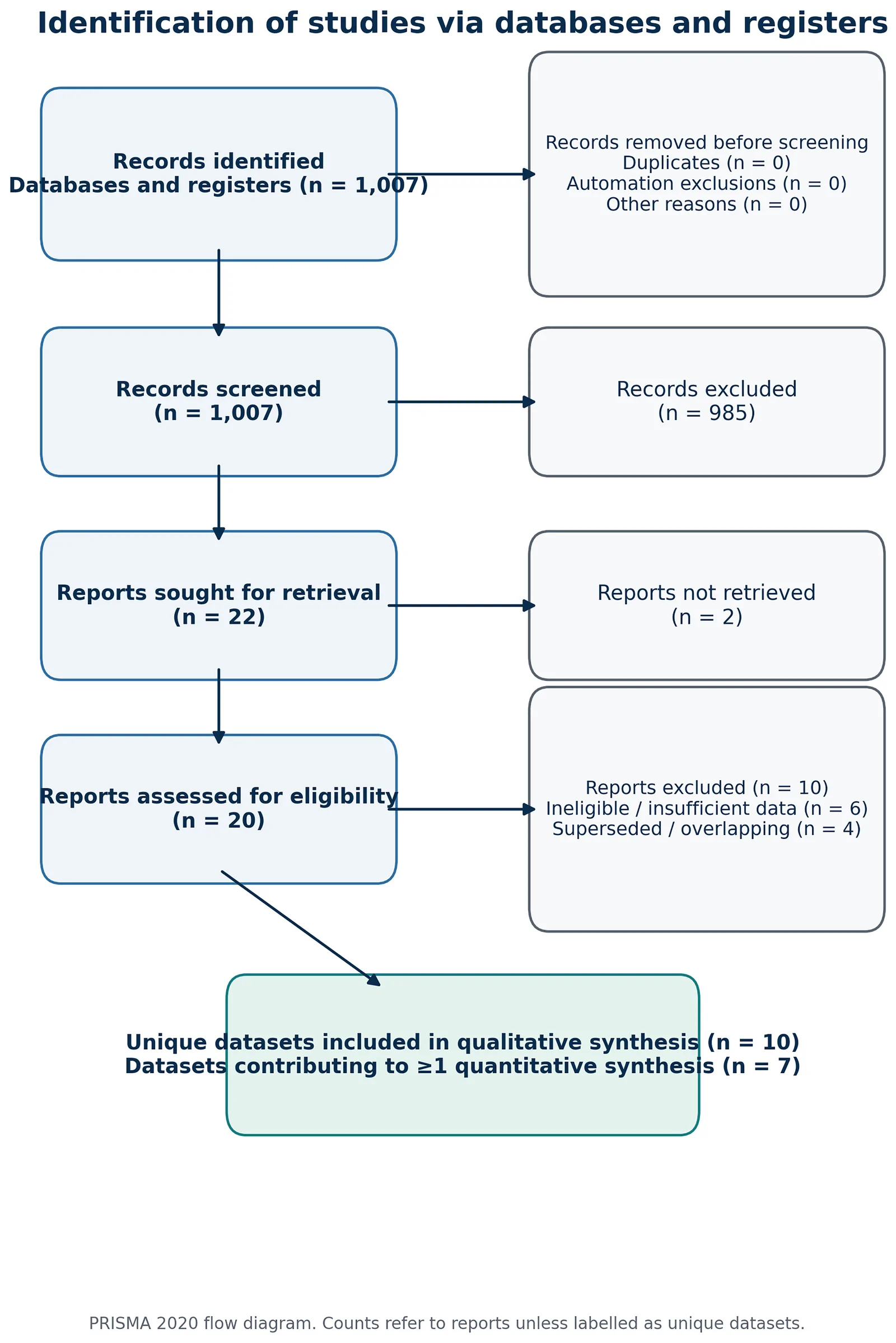
PRISMA 2020 flow diagram of study identification and selection.

Titles and abstracts were screened against the protocol criteria. Full texts were retrieved for potentially relevant reports. When overlapping institutional cohorts were identified, the most complete or most recent report was used for quantitative synthesis and earlier reports were retained only as contextual or superseded evidence. Reasons for exclusion were documented. The final study-flow numbers are shown in **Figure 1**.

### Data extraction

A standardized extraction form captured study design, country and period, sample size, patient selection, emphysema severity, baseline pulmonary function, cancer stage and histology, surgical approach, extent of cancer resection, LVRS technique, comparator details, perioperative mortality, postoperative complications, lung function, exercise capacity, dyspnoea, quality of life, recurrence, and survival. Outcome definitions were checked against the source reports and against the synthesis decision log before pooling (**Supplementary File 3**).

### Risk of bias and certainty of evidence

Uncontrolled case series were appraised using the JBI case-series domains (19). Non-randomized comparative studies were appraised with ROBINS-I (20), and the randomized clinical study was appraised using RoB 2 principles (21). Certainty of evidence for key outcomes was judged using GRADE (22), considering risk of bias, inconsistency, indirectness, imprecision, and publication bias.

### Data synthesis

Quantitative synthesis was undertaken only when at least three studies reported comparable outcome data. Single-arm proportions were logit transformed and pooled using DerSimonian-Liard random-effects model. Because mortality analyses contained zero-event studies, 0.5 was added to both event and non-event cells in every contributing study; this permits inclusion of zero-event studies but can separate the model estimate from the crude rate when events are sparse. Study markers in the forest plots show observed proportions with Wilson 94% Cis, while the pooled diamond shows the model estimate.

FEV1 change was pooled as mean difference in litres. When the standard deviation of change was not reported, it was estimated from preoperative and postoperative standard deviations using an assumed within-person correlation of 0.5; sensitivity analyses tested correlations of 0.25 and 0.75. Comparative meta-analysis was not performed because fewer than three comparable comparative datasets were available for any outcome. Survival was summarized narratively because hazard ratios, standard errors, and number-at-risk data were not consistently available. Funnel plots and Egger tests were not performed because no pooled analysis included ten or more studies.

All analyses and figure were produced in Python using NumPy, SciPy, pandas, and Matplotlib. Sensitivity analyses comprised lower-risk-of-bias restriction, a broader early-mortality definition, broader airleak definitions, strict confirmed malignancy restriction, and alternative paired-correlation assumptions. The executable analysis code is provided as **Supplementary Material**.

## Results

### Study selection

The search identified 1,007 records. All 1,007 records were screened at title/abstract stage, of which 985 were excluded. Twenty-two reports were sought for full-text retrieval; two could not be retrieved. Twenty reports were assessed in full text. Ten unique datasets were included in the qualitative synthesis, and seven contributed to at least one quantitative synthesis. Ten assessed reports were excluded because they were ineligible for the review question, contained insufficient extractable data, or represented overlapping/superseded cohorts. **Figure 1** illustrates the study selection flowchart.

### Characteristics of included studies

The included evidence base was clinically heterogeneous and dominated by early specialist-centre surgical experience. Studies differed in surgical era, emphysema morphology, cancer stage, operation type, use of VATS, extent of nodal staging, and whether the LVRS effect was produced by formal LVRS or by resection of a poorly perfused emphysematous target lobe. Sample sizes ranged from 6 to 29 patients for most included datasets. Tang et al. was the only randomized clinical study, but it included both pulmonary and oesophageal neoplasms; only the pulmonary-neoplasm subgroup was used when compatible (10). This subgroup extraction was *post-hoc* relative to the original trial design and was not pre-specified by the trial authors; the data used from Tang et al. should therefore be interpreted with the additional caution applicable to *post-hoc* subgroup analyses (10). The characteristics of included studies are summarised in **Tables 1**, **2** presents risk of bias.

**Table 1:** Characteristics and synthesis role of included unique datasets.

Study	Design/cohort	Intervention category	Procedure	Synthesis role
McKenna et al., 1996 (6)	Case series, USA LVRS programme; n=11 suspected NSCLC	Formal LVRS + cancer resection	VATS wedge resection (n=8) or lobectomy (n=3) with unilateral or bilateral LVRS	Primary mortality and air-leak synthesis
Choong et al., 2004 (2)	Retrospective review; USA; n= 21 lung cancers	Mixed: lobar effect/formal LVRS	Lobectomy in destroyed lobe (n=9), or lobectomy/wedge plus LVRS (12)	Primary mortality, air-leak, FEV1 and survival synthesis
DeRose et al., 1998 (7)	Case series; USA; 14 patients; 16 lesions; 9 NSCLC	Formal LVRS + nodule resection	Wide wedge (n=11) or lobectomy (n=3)	Primary mortality, air-leak and FEV1 synthesis
Ojo et al., 1997 (11)	Comparative study; USA; n=11; 3 cancers	Formal LVRS + nodule resection	LVRS target resection+/- minimal wedge; non-comparable lobectomy controls	Narrative/safety only
Edwards et al., 2001 (8)	Cohort; UK; n=29 NSCLC; high-risk subgroup n=14	Lobar volume-reduction effect	Anatomical lobectomy producing lobar volume-reduction effect	Mortality only; no causal comparison
Pompeo et al., 2003 (3)	Retrospective stage I cohort; Italy; n=16	Mixed: formal/sublobar/lobar	LVR alone (n=5), wedge (n=3), segmentectomy (n=2), lobectomy (n=6); bilateral LVR (n=5)	Primary mortality, air-leak, FEV1, survival and QoL synthesis
Teschner et al., 2003 (13)	Case series; Germany; n=6 NSCLC	Mixed: formal/LVRS-like concept	Lobectomy (n=4) or extra-anatomical resection (n=2) with simultaneous LVRS concept	Narrative only/high risk of bias
Hayashi et al., 1999 (14)	Case series; Japan; n=10 lung cancers	Mixed: sublobar/lobar effect	Lobectomy (n=3) or partial resection (n=7); VATS (n=5)	Review plus sensitivity/narrative; excluded from primary endpoints
Caviezel et al., 2018 (4)	Database review; Switzerland; n=14	LVRS-like sublobar resection.	Atypical resection (n=10) or segmentectomy (n=4) in LVRS concept; unilateral/bilateral; VATS/open	Primary mortality, air-leak, FEV1 and survival synthesis
Tang et al., 2009 (10)	Randomized study; China; A n=23/B n=22	Formal LVRS + cancer resection	Tumour resection plus same-side LVRS vs tumour resection alone	Functional/safety synthesis

AF, atrial fibrillation; FEV1, forced expiratory volume in 1 second; LOS, length of stay; LVRS, lung volume reduction surgery; NSCLC, non-small-cell lung cancer; PAL, prolonged air leak; ppoFEV1, predicted postoperative FEV1; QoL, quality of life; 6MWD, 6-minute walk distance.

**Table 2:** Risk-of-bias summary and use in quantitative synthesis.

Study	Tool	Overall judgement	Primary quantitative use	Main reason for judgement
Choong et al., 2004 (2)	JBI case series	Low	Yes	Updated Barnes cohort; clear outcomes and complete follow-up
McKenna et al., 1996 (6)	JBI case series	Low	Yes	Clear denominator and perioperative/functional outcomes
DeRose et al., 1998 (7)	JBI case series	Low	Yes	Clear inclusion/exclusion criteria and objective follow-up
Pompeo et al., 2003 (3)	JBI case series	Low	Yes	Well-described stage I cohort with repeated QoL/functional outcomes
Caviezel et al., 2018 (4)	JBI case series	Low	Yes	Database search, selection criteria, mortality and FEV1 clearly reported
Hayashi et al., 1999 (14)	JBI case series	Moderate	Sensitivity/narrative	Small mixed-stage series and incomplete compatible pulmonary-function reporting
Teschner et al., 2003 (13)	JBI case series	High	Narrative only	Small sample and limited sampling/completeness/statistical reporting
Ojo et al., 1997 (11)	ROBINS-I	Serious	Narrative/safety only	Controls differed substantially in baseline lung function and indication; mostly benign nodules
Edwards et al., 2001 (8)	ROBINS-I	Serious for causal comparison	Objective group-specific mortality only	Risk strata were not treatment comparisons; confounding by indication
Tang et al., 2009 (10)	RoB 2 principles	Some concerns	Pulmonary subgroup cautiously	Randomization details and reporting unclear; mixed tumour population

JBI, Joanna Briggs Institute; ROBINS-I, Risk Of Bias In Non-randomized Studies of Interventions; RoB 2, revised Cochrane risk-of-bias tool for randomized trials.

### Intervention categories

Formal concomitant LVRS. Predominated in McKenna, Derose, Ojo, and Tang, with additional formal-LVRS patients in Choong and Pompeo. LVRS-like sublobar target resections predominated in Caviezel, and were used in Teschner, Hayashi, and Pompeo. Edwards evaluated lobectomy in a poorly perfused target lobe, while Choong and Pompeo also contained lobar-effect procedures. Outcomes were rarely stratified by category within mixed datasets; category-specific meta-analysis would therefore have required double counting or unsupported allocation.

### Risk of bias

Overall risk of bias was important. Selection bias was inherent because most reports enrolled patients considered favourable for LVRS anatomy, cancer resectability, and specialist perioperative care. Objective outcomes such as mortality and FEV1 were more reliable than complications, survival, or recurrence, where definitions and follow-up varied. **Table 2** summarises the risk of bias assessment.

### Perioperative mortality

**Figure 2:**
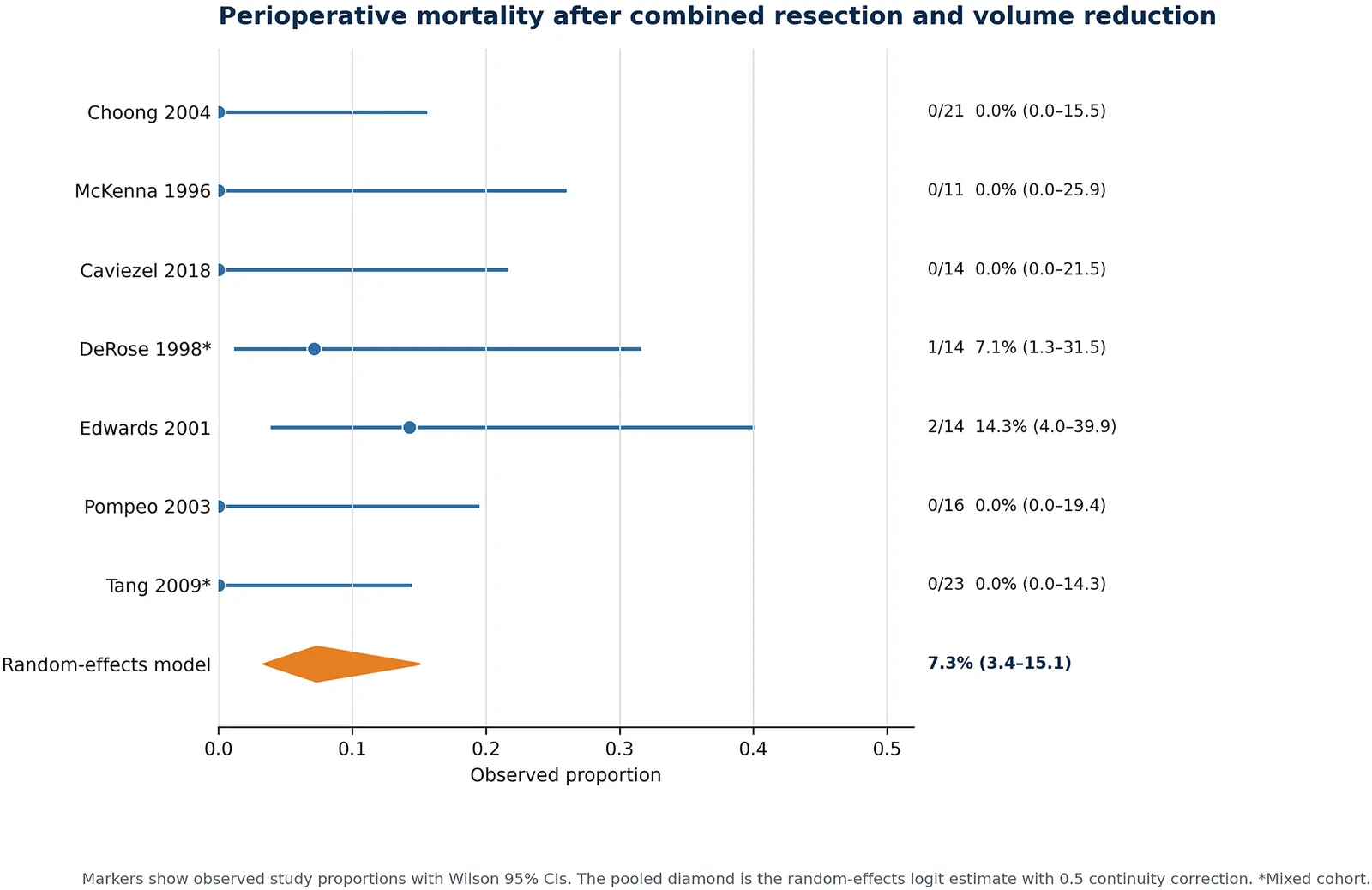
Perioperative mortality after combined lung cancer resection and lung volume reduction. Markers represent observed study proportions with Wilson 95% confidence intervals (CIs), and the orange diamond represents the random-effects pooled estimate. The continuity-corrected random-effects logit model estimated perioperative mortality at 7.3% (95% CI 3.4–15.1). The asterisk denotes a mixed cohort.

Seven studies including 113 patients contributed to the primary mortality synthesis (2–4, 6–8, 10). The pooled perioperative mortality estimate was 7.3% (95% CI 3.4-15.1; I2 = 0%). However, the directly observed crude event rate was 3/113 (2.7%), so the pooled estimate should be interpreted as the output of a sparse-event logit-proportion model rather than as the observed mortality rate. The apparent I^2^ of 0% should not be interpreted as evidence of true clinical homogeneity: five of seven studies reported zero perioperative deaths, and with only three observed deaths overall the analysis has very limited power to detect heterogeneity. In a lower-risk-of-bias sensitivity analysis restricted to five studies and 76 patients, the pooled estimate was 5.1% (95% CI 1.8-13.6). In the strict confirmed-malignancy sensitivity analysis, 2/76 deaths were observed (2.6%), and the model estimate was 7.6% (95% CI 3.0–18.0). Leave-one-out estimates ranged from 4.5% to 8.2%. Adding the 2-month death from Hayashi et al. in a broader early-mortality sensitivity analysis produced a similar estimate of 8.2% (95% CI 4.1-15.7) (14). **Figure 2**; **Table 3** present both observed and model-based results.

**Table 3:** Quantitative synthesis and sensitivity analyses.

Outcome	Analysis set	Studies/participants	Events or data	Pooled estimate (95% CI)	I2	Interpretation
Perioperative mortality	Primary single-arm	7/113	3 deaths	7.3% (3.4-15.1)	0%	Acceptable but non-negligible mortality in selected patients
Perioperative mortality	Lower-risk-of-bias sensitivity	5/76	1 death	5.1% (1.8-13.6)	0%	Estimate remains low but imprecise
Early mortality	Including Hayashi 2-month death	8/123	4 deaths	8.2% (4.1-15.7)	0%	Broader definition does not materially alter inference
Prolonged air leak	Primary single-arm	5/76	28 events	38.0% (26.9-50.6)	16.3%	Dominant morbidity signal
Any/unspecified air leak	Sensitivity	7/128	44 events	35.7% (27.9-44.3)	0%	Similar estimate despite broader definition
FEV1 change	Exploratory pre-post	4/74	Continuous	+0.26 L (+0.20 to +0.32)	0%	Supports volume-reduction physiological effect
Recurrence	Exploratory single-arm	4/58	12 events	25.1% (15.4-38.2)	Not estimable (sparse events; wide CI; high definitional heterogeneity)	Very uncertain due to follow-up and staging heterogeneity

FEV1, forced expiratory volume in 1 second; I2, percentage of variability attributable to heterogeneity; LVRS, lung volume reduction surgery.

### Postoperative morbidity and prolonged air leak

**Figure 3:**
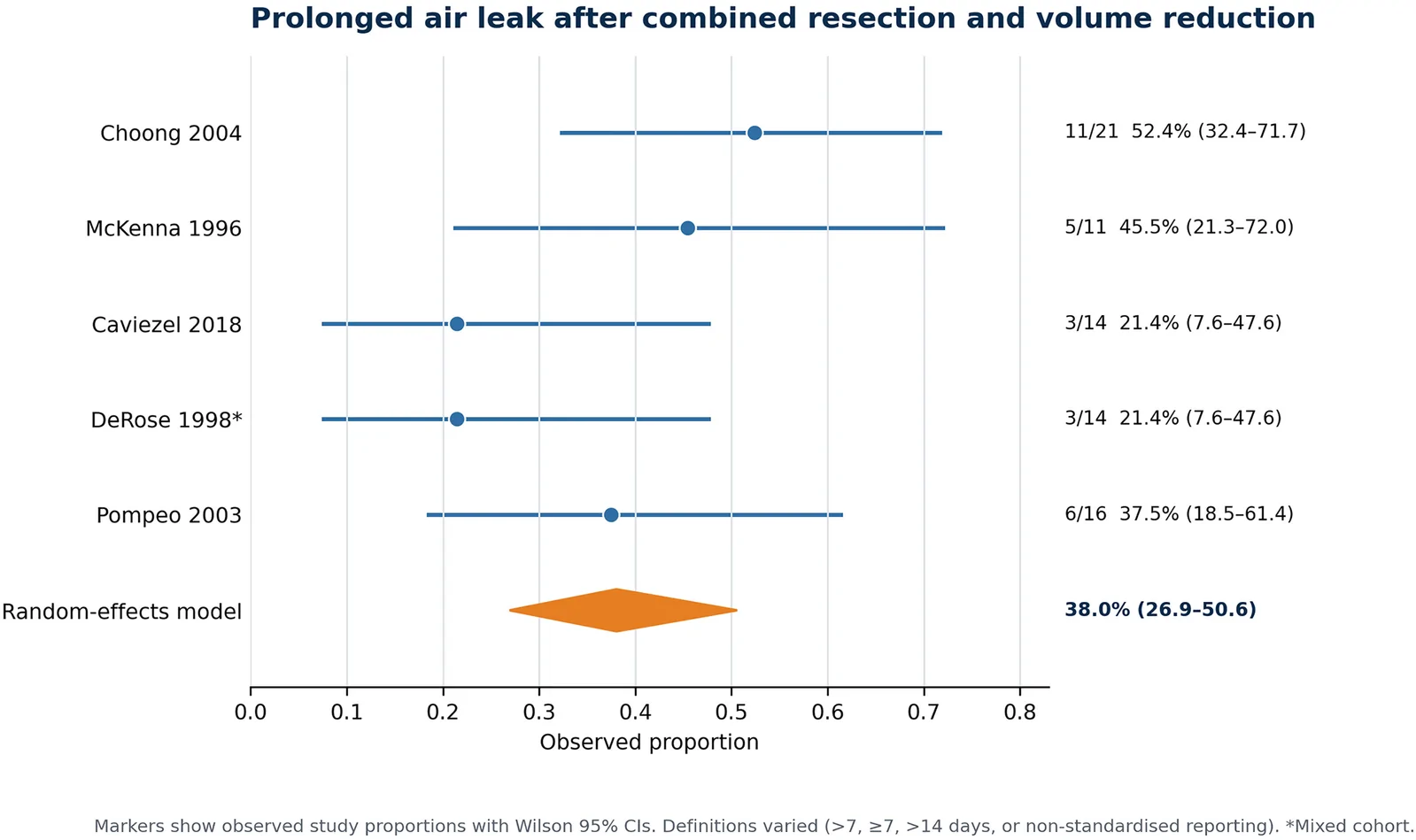
Prolonged air leak after combined lung cancer resection and lung volume reduction. Markers represent observed study proportions with Wilson 95% CIs, and the orange diamond represents the random-effects pooled estimate of 38.0% (95% CI 26.9–50.6). Definitions varied across studies, including thresholds of >7, ≥7, or >14 days and non-standardised reporting. The asterisk denotes a mixed cohort.

Prolonged air leak was the most consistently reported complication. Five studies including 76 patients contributed 28 events, pooled estimate was 38.0% (95% CI 26.9-50.6; I2 = 16.3%) (2–4, 6, 7). The result was stable in a broader sensitivity analysis that included any or unspecified air leak (35.7%, 95% CI 27.9-44.3; seven studies, 128 patients). The pooled 38% estimate must be interpreted in the context of substantial definitional heterogeneity across contributing studies (thresholds varied between >7, ≥7, >14 days, and non-standardised reporting). Excluding the DeRose mixed cohort yielded 25/62 events and a pooled estimate of 41.5% (95% CI 29.8–54.3); leave-one-out estimates ranged from 32.9% to 41.5%. **Figure 3**; **Table 3** provide the analyses. Other complications were variably reported and included atrial fibrillation, pneumothorax after chest-tube removal, respiratory failure or reintubation, bronchopneumonia, bleeding requiring re-exploration, and reoperation for persistent fistula or air leak.

### Pulmonary function and symptom outcomes

**Figure 4:**
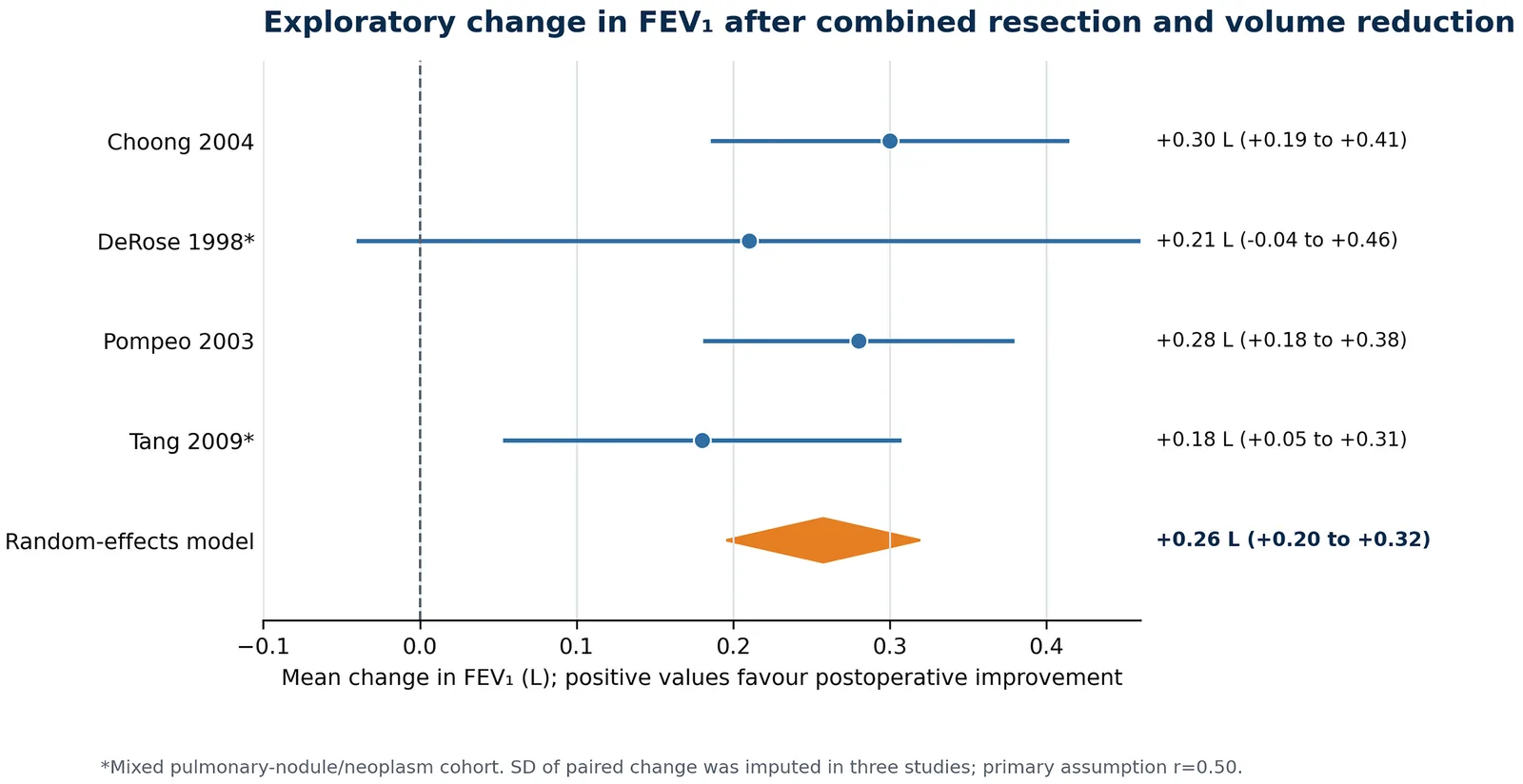
Exploratory change in forced expiratory volume in 1 second (FEV₁) after combined lung cancer resection and lung volume reduction. Positive values indicate postoperative improvement. The random-effects pooled mean change was +0.26 L (95% CI +0.20 to +0.32). Standard deviations of paired changes were imputed for three studies using an assumed within-person correlation of 0.50. The asterisk denotes a mixed pulmonary-nodule/neoplasm cohort.

Four studies including 74 patients reported FEV1 change in litres sufficiently consistently for exploratory pooling (2, 3, 7, 10). The pooled mean FEV1 increase was +0.26 L (95% CI + 0.20 to +0.32) (**Figure 4**), and results remained +0.25 to +0.26 L across correlation assumptions and +0.24 to +0.28 L in leave-one-out analyses. Three of the four contributing studies required SD imputation using the assumed within-person correlation of 0.5; this estimate is therefore model-dependent and should be interpreted with caution. The absence of a concurrent control arm means that improvement cannot be attributed causally to the combined procedure — observed gains may reflect patient selection (only survivors with complete follow-up contributed data), spontaneous postoperative trajectory, or regression to the mean. The direction of effect is consistent with the physiological rationale, but the certainty of the FEV1 finding is very low.

Narratively, McKenna et al. reported mean FEV1 improvement from 654 mL to 1,079 mL after combined LVRS and clinical stage I cancer resection (6). Pompeo et al. reported FEV1 improvement of +0.28 L, residual volume reduction of 1.18 L, 6-minute walk distance improvement of 86 m, and sustained SF-36 improvements in selected domains for up to 24–36 months (3). Caviezel et al. reported median FEV1% improvement from 32.5% to 37% at 3 months (4). Tang et al. found significant postoperative improvements in the combined LVRS group, whereas tumour resection alone was unchanged or worsened (10).

### Oncologic outcomes and survival

Oncologic outcomes were incompletely reported and were not suitable for definitive comparative inference. Four studies with 58 patients contributed 12 recurrence events (20.7% observed) and a pooled proportion of 25.1% (95% CI 15.4-38.2) (2, 3, 6, 14). This estimate is fragile because follow-up duration, stage distribution, surgical margins, nodal evaluation, and resection type varied markedly. **Table 4** summarizes stage, histology, resection, margins, nodal assessment, recurrence, follow-up, and survival for every included dataset.

**Table 4:** study level characteristics and outcomes.

Study	Stage/Histology	Resection	Margins	Nodal assessment	Recurrence	Follow-up	Survival
McKenna et al., 1996 (6)	Clinical I (11); pathological I (10), III/N2 (1); squamous (7), adenocarcinoma (4)	Wedge 8; lobectomy 3	NR	Lymph-node dissection reported	0 recurrences; NED defined clinically	Mean 9.7 months (2–20)	10 alive NED; 1 pulmonary-embolus death NED
Choong et al., 2004 (2)	I 16; II 2; III 2; IV 1; adenocarcinoma 11, squamous 8, poorly differentiated NSCLC 2	Lobectomy 18; wedge 3; additional LVRS in 12	One unsuspected positive parenchymal margin	Mediastinoscopy 15; one positive hilar node	5/21; first detected at 7–48 months; local/systemic confirmation not standardized	Mean 3.9 years; minimum 1 year; complete	OS 100%, 73.7%, 62.7% at 1, 3, 5 years
DeRose et al., 1998 (7)	9 NSCLC among 16 lesions: squamous 4, bronchioloalveolar 3, adenocarcinoma 2; stage NR	Wide wedge 11; lobectomy 3 (mixed cohort)	Grossly clear in all; one microscopic vascular/lymphatic invasion at margin	No routine mediastinal/hilar sampling	One mediastinal recurrence at 12 months among two bronchioloalveolar cancers	Mean 22.6 months (12–35)	One postoperative death; remaining patients alive/well at report
Ojo et al., 1997 (11)	3/11 malignant: adenocarcinoma, squamous, large-cell; stage NR	Target resection ± minimal wedge	NR	NR	No cancer recurrence reported	Mean 13 months	Cancer-specific survival NR
Edwards et al., 2001 (8)	I 15; II 9; IIIA 4; IIIB 1; NSCLC histology details NR	Anatomical lobectomy	NR	Nodal assessment NR in extract	Recurrence definition/events NR in index report	Median 12 months	No median-survival difference between ppoFEV_1_ strata; stage predicted 6-month survival
Pompeo et al., 2003 (3)	Pathological stage I NSCLC	LVR alone 5; wedge 3; segmentectomy 2; lobectomy 6	NR	Radical lymphadenectomy; no positive nodes	4/16 reported; definition/time-to-event not standardized	Functional/QoL follow-up to 24–36 months	Actuarial 5-year OS 68%
Teschner et al., 2003 (13)	I 4; II 1; IIIA 1; NSCLC	Lobectomy 4; extra-anatomical 2	NR	NR	Recurrence reporting non-standardized; concern concentrated after limited resection	Up to 56 months	OS 66% at 2 years; 34% at 3 years
Hayashi et al., 1999 (14)	IA 4; IB 3; IIA 1; IIIA 1; IIIB 1; adenocarcinoma 5, squamous 4, large-cell 1	Lobectomy 3; partial resection 7	NR	NR	3/10: one resection-site and two other-site recurrences	9–59 months	Eight alive at report; deaths at 2 and 17 months were non-cancer causes
Caviezel et al., 2018 (4)	IA 11; IB 1; IV 1; sarcoma unstaged; adenocarcinoma 7, large-cell 3, squamous 3, sarcoma 1	Atypical 10; segmentectomy 4 + LVRS	NR	Systematic nodal dissection in 2	Recurrence definition/events NR	6–126 months	OS 50% at 3 years; 36% at 5 years
Tang et al., 2009 (10)	Pulmonary neoplasms; stage and histology NR	Lobectomy/tumour resection ± ipsilateral LVRS	Final margin status NR	Lymph-node removal reported	NR	6–12 months	Cancer-specific survival NR

NED, no evidence of disease; NR, not reported; NSCLC, non-small-cell lung cancer; OS, overall survival; ppoFEV₁, predicted postoperative FEV₁. Values are reported as presented in source publications; heterogeneous definitions preclude direct comparison.

**Figure 5:**
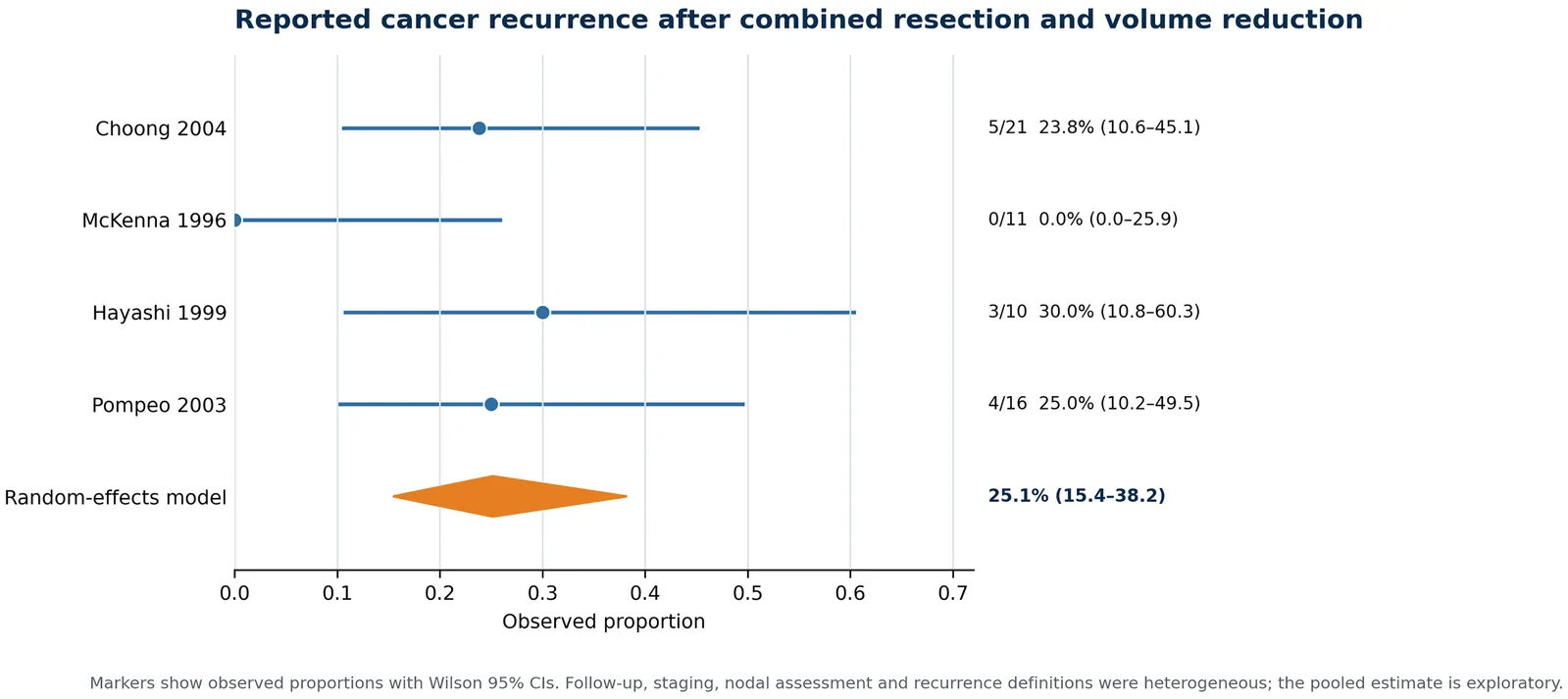
Reported cancer recurrence after combined lung cancer resection and lung volume reduction. Markers represent observed study proportions with Wilson 95% CIs, and the orange diamond represents the exploratory random-effects pooled estimate of 25.1% (95% CI 15.4–38.2). Follow-up duration, staging, nodal assessment, and recurrence definitions were heterogeneous across studies.

Survival was summarized narratively. Choong et al. reported 1- and 5-year survival of 100% and 62.7% (2). Pompeo et al. reported actuarial 5-year survival of 68%, not significantly different from an LVRS-only cohort treated during the same period (3). Caviezel et al. reported 3- and 5-year survival of 50% and 35% after sublobar resection in an LVRS concept (4). Martin-Ucar et al., a contextual long-term cohort of lobar volume-reduction-effect upper lobectomy, found 5-year survival of 35% in patients with severe heterogeneous emphysema and ppoFEV1 <40% compared with 65% in controls, while recurrence rates were similar; this suggests that long-term survival may be limited more by physiological reserve than by tumour recurrence (5). **Figure 5** demonstrates the cancer recurrence rates.

### Quantitative synthesis summary

The quantitative synthesis is detailed in **Table 3**.

### Narrative synthesis

**Table 5** synthesizes the evidence based on clinical theme.

**Table 5:** Narrative synthesis by clinical theme.

Clinical theme	Consistent finding	Evidence basis	Interpretation for practice
Patient selection	Benefit was concentrated in highly selected patients with severe heterogeneous emphysema, hyperinflation, target areas, and tumours in or near poorly functioning lung.	Choong et al., DeMeester et al., McKenna et al., Pompeo et al., Caviezel et al., Edwards et al. (1–4, 6, 8)	Use CT morphology, perfusion assessment, pulmonary rehabilitation response, and MDT judgement rather than spirometry alone.
Extent of resection	Anatomical resection was feasible when the involved lobe was emphysematous and poorly perfused; sublobar resection was used when reserve was marginal or tumours were peripheral.	McKenna et al., Choong et al., Pompeo et al., Caviezel et al., Edwards et al. (2–4, 6, 8)	Oncologic adequacy must be balanced against functional reserve; nodal staging should be optimized where safe.
Physiological effect	FEV1, dyspnoea, exercise capacity, and quality of life often improved after combined surgery.	McKenna et al., DeRose et al., Pompeo et al., Tang et al., Caviezel et al. (3, 4, 6, 7, 10)	The operation is most defensible when resection is expected to act as therapeutic volume reduction.
Morbidity	Air leak was common and more consistently reported than other complications.	McKenna et al., Choong et al., DeRose et al., Pompeo et al., Caviezel et al. (2–4, 6, 7)	Preoperative counselling should emphasize prolonged drainage and the need for specialist air-leak management.
Oncologic uncertainty	Survival varied and recurrence reporting was inconsistent; sublobar strategies may risk local recurrence in some series.	Teschner et al., Hayashi et al., Choong et al., Pompeo et al., Caviezel et al., Martin-Ucar et al. (2–5, 13, 14)	The evidence supports feasibility, not equivalence to standard oncologic surgery or superiority over SABR/SBRT.
Comparative evidence	The randomized and comparative data were not sufficiently comparable for pooled effect estimates.	Tang et al., Ojo et al., Edwards et al. (8, 10, 11)	Future studies should compare combined surgery with SABR/SBRT, standard sublobar resection, or non-operative management using adjusted designs.

MDT, multidisciplinary team; SABR/SBRT, stereotactic ablative/stereotactic body radiotherapy.

## Discussion

### Principal findings

This review suggests that lung cancer resection combined with LVRS or a volume-reduction concept is feasible in highly selected patients with severe emphysema. The model-based pooled perioperative mortality estimate was 7.3%, but the directly observed perioperative death rate in the primary analytic set was only 3/113 (2.7%); therefore, mortality should be described as low in observed terms but imprecisely estimated by sparse-event meta-analysis. The major morbidity signal was prolonged air leak, affecting approximately 38% of patients. Importantly, pooled FEV1 change favoured improvement rather than deterioration, with a mean gain of 0.26 L.

This functional finding is the central clinical rationale for combined surgery. Standard lung resection removes functioning lungs and is expected to reduce FEV1. In contrast, the combined LVRS strategy deliberately removes hyperinflated, poorly perfused parenchyma. When the tumour is located within the most destroyed lobe, or when a cancer resection can be supplemented by LVRS in a target area, the volume-reduction effect may offset the functional cost of cancer surgery and may even improve postoperative mechanics (2–4, 6, 8, 10). However, the umbrella synthesis should not be interpreted as evidence that formal LVRS, LVRS-like sublobar resection, and lobar volume-reduction-effect resection are equivalent. They were considered together only for short-term feasibility, safety, and functional synthesis because each was undertaken with a planned LVR physiological intent. Procedure-specific effects could not be isolated. Resection type remains clinically important for margins, nodal assessment, local control, and long-term survival; these oncologic outcomes were therefore not pooled by procedure and remain narrative. Procedural heterogeneity was treated as indirectness and contributes to the very-low certainty rating. Additionally, the scope of the review include perioperative outcomes which would be comparable between malignant and non-malignant tumours from an operation feasibility perspective.

### Interpretation of perioperative risk

The procedure should not be interpreted as low risk. The patients in these studies had severe physiological limitations and would often have been considered inoperable by conventional criteria. The relevant clinical comparison is therefore not routine lobectomy in fit patients, but alternative management in physiologically marginal patients, including limited resection, radiotherapy, ablation, or no definitive local therapy (1, 4, 5).

The apparent safety in specialist centres should also not be generalized to unselected COPD populations. Nearly all included cohorts used strict selection for favourable LVRS anatomy, resectability, absence of prohibitive nodal disease, and ability to tolerate thoracic surgery (1–4, 8). Centres performing combined procedures also had expertise in pulmonary rehabilitation, lung-volume-reduction selection, emphysema imaging, ventilation/perfusion interpretation, air-leak management, and high-dependency postoperative care.

### Air leak and recovery burden

Prolonged air leak (PAL) occurred in a pooled 38% of patients (2–4, 6, 7). The mechanism is the fragility of emphysematous lung parenchyma. Chronic alveolar destruction produces enlarged airspaces with thinned, avascular septa that possess minimal tensile strength and poor healing capacity. Staple lines applied to such tissue are susceptible to micro-disruption, incomplete compression, and delayed epithelialisation. Hyperinflation imposes continuous outward tension on staple lines and residual lung, further promoting leaks. Additional risk factors include coexistent bullae, subpleural blebs, peritumoural inflammation or fibrosis, and the creation of new pleural surfaces or residual spaces after resection. Buttressing, sealants, and pleural tents were variably used but cannot be compared from these data. Future studies should use standard postoperative-day threshold and report drain duration and reintervention.

### Postoperative respiratory status and ventilatory management

Postoperative respiratory failure, reintubation, and ventilator duration were reported inconsistently and could not be pooled. This is an important evidence gap in patients with limited reserve. Evidence from pneumonectomy and other extensive lung resections is indirect but clinically informative: postoperative pulmonary complications were strongly associated with in-hospital mortality, especially early ARDS (23–25).

Contemporary perioperative management favours lung-protective one-lung ventilation using low tidal volumes based on predicted body weight, avoidance of excessive airway and driving pressures, individualised positive end-expiratory pressure, judicious recruitment and avoidance of unnecessary hyperoxia; no single setting is universally optimal (26, 27). In a multicentre randomised trial, Piccioni et al. found similarly low ARDS rates with two one-lung ventilation bundles and no clear superiority for a 4-mL/kg strategy over 6mL/kg, reinforcing individualised rather than formulaic ventilation (26). After surgery, early recognition of atelectasis, pneumonia, air leak, fluid overload, and respiratory fatigue is essential. High flow oxygen, non-invasive support, or invasive ventilation should be individualised; if ARDS develops, standard lung-protective ventilation with pressure limitation and careful fluid management is appropriate. No included study directly compared ventilatory strategies after the combined procedure.

### Functional implications

The pooled FEV1 improvement supports the view that predicted postoperative FEV1 calculations can be misleading in heterogeneous emphysema (2, 5, 8). Segment-counting methods assume each resected segment contributes equally to function. In LVRS candidates, however, a tumour-bearing lobe may contribute little effective ventilation or perfusion and may worsen mechanics through hyperinflation. Preoperative CT and perfusion imaging are therefore central to identifying whether resection is likely to remove a useful lung or a physiological target area.

**Figure 6:**
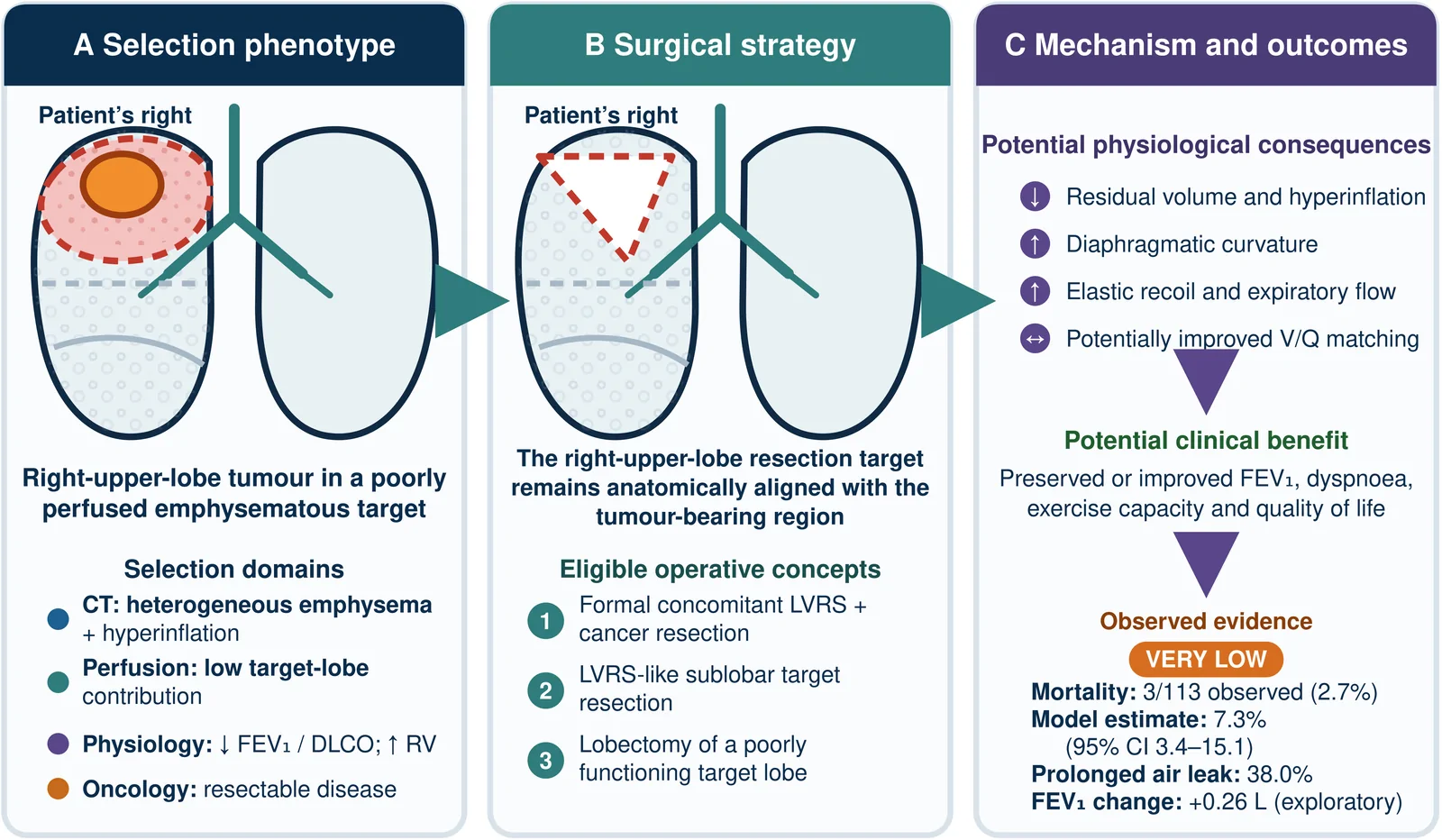
Physiological rationale and proposed framework for combined lung cancer resection and lung volume reduction. **(A)** Selection phenotype in which a resectable tumour lies within poorly perfused emphysematous lung, assessed using CT morphology, regional perfusion, physiological reserve, and oncologic resectability. **(B)** Operative strategies comprising formal concomitant LVRS and cancer resection, LVRS-like sublobar target resection, or lobectomy of a poorly functioning target lobe. **(C)** Proposed physiological consequences, potential clinical benefits, and the principal pooled outcomes. The certainty of the observed evidence is very low. CI, confidence interval; CT, computed tomography; DLCO, diffusing capacity of the lung for carbon monoxide; FEV₁, forced expiratory volume in 1 second; LVRS, lung volume reduction surgery; RV, residual volume; V/Q, ventilation–perfusion.

The physiological mechanisms underlying this paradoxical improvement are those established for LVRS in severe emphysema and are supported by the functional patterns reported in the included surgical cohorts (1–4, 6, 8, 9, 28). Hyperinflated, poorly compliant lung regions exhibit loss of elastic recoil, premature airway closure, ventilation-perfusion inefficiency, and mechanical disadvantage to the diaphragm due to caudal displacement and flattening. When the tumour lies within a poorly perfused emphysematous target lobe, or when cancer resection can be anatomically combined with resection of a separate LVRS target area without sacrificing essential functional parenchyma, the reduction in residual volume and improvement in chest-wall and diaphragmatic mechanics can offset the functional cost of resection and may produce a net FEV1 gain. This contrasts with standard lobectomy in patients without favourable LVRS anatomy, where removal of functional lung units would be expected to reduce postoperative spirometry. The combined operation should therefore be framed as a dual therapeutic strategy only when preoperative CT, perfusion assessment, TLCO/FEV1, contralateral-lung status, oncologic resectability, and MDT judgement confirm that the planned resection removes lung that is contributing minimally to gas exchange while impairing global mechanics. **Figure 6** illustrates the physiological rationale for the combined procedure.

### Proposed patient-selection and staging framework

The extreme selection in the source cohorts is not merely a limitation; it defines the circumstances in which the physiological premise is plausible. Because the evidence is observational and no selection rule has been prospectively validated, the following framework is a pragmatic multidisciplinary checklist rather than a graded recommendation or eligibility score.

A potential candidate should satisfy all of the following domains: (i) curative-intent oncologic resectability, including a technically feasible R0 resection and appropriate nodal assessment; (ii) a CT-defined LVRS target, ideally a markedly destroyed, hyperinflated region that contains the tumour, or a compatible separate target that can be treated without sacrificing preserved parenchyma; (iii) quantitative perfusion showing that the intended specimen contributes little regional function and that the contralateral lung has an acceptable distribution of reserve; (iv) acceptable global cardiopulmonary and frailty risk after full optimisation, based on FEV1, DLCO, split-function predicted postoperative values, gas exchange, exercise testing and, when indicated, cardiopulmonary exercise testing; and (v) treatment at an experienced thoracic/LVRS centre able to manage prolonged air leak and postoperative respiratory failure. These domains recapitulate selection in the included cohorts rather than define universal numerical cut-offs (1–4, 6, 8, 29).

For oncologic staging, contrast-enhanced CT and FDG-PET/CT should be used according to contemporary lung-cancer guidance, with brain imaging and invasive mediastinal staging when tumour or nodal findings warrant them (30). FDG-PET/CT assesses tumour and nodal or metastatic disease; it should not be conflated with quantitative perfusion or ventilation/perfusion (V/Q) imaging. The latter maps lobar contribution to function and can refine split-function estimates when standard segment counting is unreliable, but it does not stage cancer (29, 31). A PET-negative mediastinum also does not by itself obviate pathological nodal staging when tumour features indicate it and the result would change treatment.

Features arguing against combined resection–LVRS include the absence of a physiologically credible target; planned removal of functionally important parenchyma; poor distribution of contralateral function; inability to achieve an R0 resection or adequate nodal assessment; or prohibitive cardiac, gas-exchange, exercise, frailty or rehabilitation risk despite optimisation. No single FEV1, DLCO or predicted-postoperative threshold can be promoted as an absolute rule from this review.

### Oncologic interpretation

The oncologic evidence remains the weakest part of the literature. Most patients had early-stage disease, but lymph node assessment was inconsistent, and some patients underwent wedge or atypical resections because of physiological constraints (2–4, 13, 14). Survival was often reported as actuarial estimates without hazard ratios or standardized recurrence definitions. Martin-Ucar et al. is important because recurrence rates were similar between severe-emphysema and control lobectomy groups, while survival was worse in the emphysema group, supporting the interpretation that physiological reserve, not cancer recurrence alone, drives long-term outcome (5).

### Non-surgical alternatives and MDT decision making

Potential alternatives include stereotactic body radiotherapy (SBRT) or other radiotherapy techniques (including proton therapy where relevant), image-guided thermal ablation, and stage-appropriate chemotherapy or immunotherapy (30, 32–37). However, none of the included studies directly compared any of these strategies with combined resection–LVRS. They are therefore added as alternatives. They are therefore acknowledged here as alternatives rather than reviewed in detail.

Radiotherapy may be unsuitable in some patients because of tumour location, achievable lung-dose constraints, or concern that pneumonitis or even a small functional loss would be poorly tolerated by a frail emphysematous lung. Poor baseline FEV1 alone is not a validated absolute exclusion from SBRT, but extreme respiratory limitation must be weighed in the individual radiotherapy plan (32, 33). Histologic confirmation is recommended whenever feasible; however, percutaneous biopsy through markedly emphysematous parenchyma increases pneumothorax and chest-drain risk, while chemotherapy or immunotherapy generally depends on tissue diagnosis and biomarker characterisation. If biopsy is unsafe, empiric SBRT may still be considered after MDT consensus when imaging strongly supports malignancy (33, 38). Additional reports excluded from the primary synthesis, superseded by larger reports, or retained only as contextual evidence are detailed in **Supplementary Table 1** (39–42). If non-surgical treatment is nevertheless unsafe, unsuitable, or unlikely to be curative, resection of a highly suspicious lesion within a poorly functioning LVRS target may be the only potentially definitive option and may also provide a volume-reduction benefit. The final decision should be made case by case in a thoracic MDT, integrating oncologic probability and stage, biopsy feasibility, radiotherapy planning, emphysema distribution and perfusion, operative risk, expected LVRS benefit, and patient preference.

## Strengths and limitations

The strengths of this review include prospective registration, explicit handling of overlapping cohorts, endpoint compatibility checks, risk-of-bias sensitivity analyses, and avoidance of inappropriate comparative pooling. The limitations are substantial: most studies were retrospective, small, single-centre, and conducted in expert LVRS units; selection bias was unavoidable; complication definitions varied; postoperative pulmonary-function data may be affected by survivorship and selective follow-up. Publication bias and positive-outcome reporting are plausible because small successful specialist-centre experiences are more likely to be reported, but formal tests were underpowered.

## Clinical implications

Combined lung cancer resection and LVRS should be considered a specialist option for carefully selected patients with severe emphysema, particularly when the tumour is located within a destroyed target area (1–4, 8). The strongest candidates are likely to have heterogeneous emphysema, marked hyperinflation, poor perfusion of the tumour-bearing region, no prohibitive nodal or metastatic disease, and capacity to complete pulmonary rehabilitation. The findings do not support indiscriminate expansion of surgery to all patients with COPD and lung cancer.

## Research implications

Future research should prioritize prospective multicentre registries and adjusted comparative studies. A minimum reporting standard for future publications is proposed below. Perioperative outcomes: 30-day mortality, 90-day mortality, in-hospital mortality, prolonged air leak (with explicit threshold in days), chest-drain duration, outpatient drain management, respiratory failure, reintubation, atrial fibrillation, and reintervention rate. Functional outcomes: FEV1, DLCO, residual volume, 6-minute walk distance, dyspnoea score, and quality of life instrument with timepoints. Oncologic outcomes: surgical margins, nodal assessment method and yield, local recurrence, distant recurrence, disease-free survival, overall survival, and competing respiratory mortality. Covariate adjustment in comparative studies should include emphysema distribution and heterogeneity, ppoFEV1, DLCO, tumour stage, resection type, and surgical approach. No ongoing prospective multicentre registries or randomized trials specifically addressing this population are known to the authors at the time of submission; this gap is the primary argument for a coordinated registry effort.

## Conclusion

In selected patients with severe emphysema and resectable lung cancer, combined lung cancer resection and LVRS appears feasible, but certainty is very low. The directly observed perioperative mortality was 3/113 (2.7%). The continuity-corrected random-effects logit estimate was 7.3% (95% CI 3.4–15.1), but this is a method-dependent pooled estimate—not the observed mortality risk—and is unstable with only three deaths. FEV1 pooling suggested an improvement of 0.26 L. Prolonged air leak is common, affecting approximately 38% of patients. The evidence supports specialist feasibility and physiological plausibility, but certainty is very low and oncologic comparative effectiveness remains unproven.

## Summary of findings and certainty of evidence

The certainty of evidence is summarised in **Table 6**.

**Table 6:** GRADE summary of findings.

Outcome	Studies/participants	Effect estimate	Certainty	Main reasons for certainty judgement
Perioperative mortality	7 studies/113 patients	7.3% (95% CI 3.4-15.1)	Very low	Observational evidence; selection bias; sparse events; imprecision
Lower-risk mortality sensitivity	5 studies/76 patients	5.1% (95% CI 1.8-13.6)	Very low	Smaller dataset; residual confounding; wide CI
Prolonged air leak	5 studies/76 patients	38.0% (95% CI 26.9-50.6)	Very low	Variable definitions; observational studies; small sample size
FEV1 change	4 studies/74 patients	+0.26 L (95% CI + 0.20 to +0.32)	Very low	Pre-post data only; no concurrent control group (indirectness); high risk of bias (survivorship and selective follow-up); serious imprecision
Recurrence	4 studies/58 patients	25.1% (95% CI 15.4-38.2)	Very low	Heterogeneous follow-up, staging and nodal assessment; small samples
Overall survival	Not pooled	Narrative synthesis only	Very low	Hazard ratios unavailable; variable survival reporting and competing mortality

CI, confidence interval; FEV1, forced expiratory volume in 1 second.

## Data Availability

The original contributions presented in the study are included in the article/**Supplementary Material**. Further inquiries can be directed to the corresponding author.

## Supplementary material

Supplementary Table 1 Reports excluded from primary synthesis, superseded by larger reports, or retained only as contextual evidence.

Supplementary File 1 Study protocol.

Supplementary File 2 Prospero entry.

Supplementary File 3 Data extraction and Risk-of-bias workbook.

Supplementary File 4 Complete database-specific search strategies.

Supplementary File 5 **(a)** Data synthesis decision log. **(b)** Analysis code. **(c)** Analysis results.
