# Supplementary material for: Outcomes of combined lung cancer resection and lung volume reduction surgery in adults with severe emphysema; a systematic review and meta-analysis
Source: Front Oncol. 2026 Aug 19;16:1916431. doi: 10.3389/fonc.2026.1916431 (PMC13533703; doi:10.3389/fonc.2026.1916431)
Supplement: Supplementary file 1 [file DataSheet1.pdf]

## Background

Lung cancer frequently coexists with advanced emphysema or chronic obstructive pulmonary disease (COPD). Patients with severe emphysema often have limited pulmonary reserve, and this may preclude definitive lung-cancer resection. Lung volume reduction surgery (LVRS) has been shown to improve symptoms and lung function in well-selected emphysema patients. Combining LVRS with lung cancer resection is therefore hypothesised to allow complete oncologic resection while providing a “lung-volume-reduction effect”; removal of hyper-expanded, non-functional lung; that stabilises or improves postoperative pulmonary function. Early case series report that lobectomy or wedge resection supplemented with LVRS can achieve acceptable morbidity and survival. However, the evidence base remains sparse and heterogeneous, and there is no consolidated synthesis of perioperative, functional and oncologic outcomes compared with standard procedures.

## Objectives

### Primary objective

1. To evaluate perioperative outcomes (30-day or in-hospital mortality and major complications) in adults with severe emphysema undergoing lung cancer resection combined with LVRS.

### Secondary objectives

1. To assess postoperative pulmonary-function changes (e.g., $FEV_1$, DLCO, residual volume) after combined surgery.
2. To summarise oncologic outcomes (overall survival, disease-free survival) of combined surgery.
3. To compare outcomes between combined surgery and:
    a. isolated lung-cancer resection,
    b. isolated LVRS,
    c. staged procedures (LVRS followed by cancer resection or vice versa),
    d. and to provide single-arm pooled estimates when comparators are absent.

## Methods

### Eligibility criteria

- Participants: Adults (≥18 years) with radiologically or histologically confirmed severe emphysema (with or without COPD) and primary lung cancer. Studies including mixed severity are eligible if data for severe emphysema can be extracted.
- Interventions: Lung cancer resection (lobectomy, segmentectomy, wedge resection or pneumonectomy) performed simultaneously with LVRS, including formal LVRS, LVRS-like resection (lobectomy acting as LVRS), or bronchoscopic/endoscopic LVRS if combined with resection.

- Comparators:
    a. Lung cancer resection alone in patients with emphysema,
    b. LVRS alone,
    c. Staged LVRS and cancer resection,
    d. No comparator (single-arm studies).
- Outcomes:
  Primary: perioperative mortality (≤30 days/in-hospital) and major postoperative complications (e.g., prolonged air leak, respiratory failure, reintubation, atrial fibrillation, pneumothorax).
  Secondary: changes in $FEV_1$ and $FEV_1$% predicted, DLCO, residual volume, exercise capacity (6-minute walk test), dyspnoea scores; length of hospital/ICU stay; oncologic outcomes (overall survival, disease-free survival, recurrence).

- Study designs: Randomised controlled trials, prospective or retrospective cohort studies, case–control studies and case series with ≥5 patients. Conference abstracts will be included if sufficient data are available. Case reports with <5 patients will be narratively summarised but excluded from quantitative synthesis.

## Information sources

Electronic searches will be conducted in:

- PubMed,
- Semantic Scholar,
- Open Alex,
- Scopus,
- Web of Science Core Collection,
- Cochrane Central Register of Controlled Trials (CENTRAL).

Additional sources:

- ClinicalTrials.gov and the WHO International Clinical Trials Registry Platform for unpublished or ongoing studies.
- Reference lists of included studies and related reviews.

## Search strategy

A comprehensive search strategy will be developed.The PubMed search will include controlled vocabulary and text-word terms relating to emphysema/COPD, lung-volume-reduction procedures and lung cancer resection. A preliminary PubMed strategy is:

((“Pulmonary Emphysema”[Mesh] OR emphysema[tiab] OR “severe emphysema”[tiab] OR “Chronic Obstructive Pulmonary Disease”[Mesh] OR COPD[tiab]))

AND

((“Lung Volume Reduction Surgery”[Mesh] OR “lung volume reduction”[tiab] OR LVRS[tiab] OR “volume reduction effect”[tiab]))

AND

((“Lung Neoplasms”[Mesh] OR “Carcinoma, Non-Small-Cell Lung”[Mesh] OR “lung cancer”[tiab] OR NSCLC[tiab]) AND (resection[tiab] OR lobectomy[tiab] OR “wedge resection”[tiab] OR surgery[tiab]))

AND

(combined[tiab] OR concomitant[tiab] OR concurrent[tiab] OR simultaneous[tiab] OR synchronous[tiab]))

Search strategies will be translated for other databases. No language restrictions will be applied. The search period will run from inception of each database to the search date (27 May 2026).

## Study records

Data management: Search results will be imported into a reference management software ( Zotero) for deduplication and subsequently into a systematic review platform for screening.

Selection process: Two reviewers will independently screen titles and abstracts. Full-text articles will be retrieved and assessed for eligibility. Disagreements will be resolved through discussion or by consulting a third reviewer. Reasons for exclusion at the full-text stage will be documented. The selection process will be presented using a PRISMA flow diagram.

Data extraction: A standardised data-extraction form will be piloted on a subset of studies. Two reviewers will extract data independently on:

- Study characteristics (authors, year, design, country, sample size).
- Participant characteristics (age, sex, smoking history, baseline pulmonary function, cancer stage and histology, emphysema distribution).
- Intervention details (type of resection, extent and technique of LVRS, surgical approach—VATS or open).
- Comparator details (where applicable).
- Outcomes as defined above (including follow-up durations).

When multiple reports of the same study exist, the most comprehensive dataset will be extracted. Authors will be contacted for missing data where feasible.

## Risk of bias assessment

Two reviewers will independently assess the risk of bias:

- For randomised controlled trials, the Cochrane Risk of Bias tool (RoB 2) will be used.
- For non-randomised studies, ROBINS-I (Risk Of Bias In Non-randomised Studies of Interventions) will be applied.

- For case series, the Joanna Briggs Institute (JBI) critical appraisal checklist for case series will be used. Disagreements will be resolved by consensus. The risk of bias will be considered when interpreting results.

## Data synthesis

Quantitative synthesis will be performed if ≥3 studies with comparable design and outcome measures are available.

### Effect measures

- Dichotomous outcomes (mortality, complications) will be pooled as risk ratios (RR) or odds ratios (OR) with 95% confidence intervals (CI). Single-arm proportions will be pooled using logit transformation.
- Continuous outcomes ($FEV_1$, DLCO, etc.) will be pooled as mean differences (MD) when measured uniformly; otherwise standardised mean differences (SMD) will be used.
- Time-to-event outcomes (overall and disease-free survival) will be summarised as hazard ratios (HR) when reported; otherwise survival proportions at common time points will be extracted.

### Model selection;

A random-effects model (DerSimonian–Laird or Restricted Maximum Likelihood) will be applied to account for heterogeneity among studies. Fixed-effect models will be used in sensitivity analyses.

### Heterogeneity assessment;

Statistical heterogeneity will be assessed using the $I^2$ statistic (values of 25%, 50% and 75% representing low, moderate and high heterogeneity) and the $\chi^2$ test ($p < 0.10$ considered significant). Sources of heterogeneity will be explored through subgroup and sensitivity analyses (see below).

## Subgroup analysis and investigation of heterogeneity

- Type of combined procedure: lobectomy alone vs lobectomy plus formal LVRS vs sublobar resection + LVRS vs bronchoscopic LVRS.
- Emphysema distribution (upper-lobe predominant vs diffuse).
- Cancer stage (early [I–II] vs advanced).
- Surgical approach (video-assisted thoracoscopic surgery vs open thoracotomy).
- Study period (pre-2005 vs 2005–2015 vs post-2015) to account for advances in technique and perioperative care.

## Sensitivity analyses

- Excluding studies at high risk of bias.
- Excluding small case series (<10 patients).
- Using alternative statistical models (fixed effect vs random effects).

- Restricting to studies reporting adjusted estimates for confounders (e.g., baseline pulmonary function).

## Assessment of reporting biases

If ≥10 studies are included in a meta-analysis, small study/publication bias will be evaluated using funnel plots and Egger's regression test. For single-arm meta-analyses, arcsine transformed plots may be used. Trim-and-fill methods will be applied to explore the impact of potential publication bias.

## Certainty of evidence

The Grading of Recommendations Assessment, Development and Evaluation (GRADE) framework will be used to assess the overall certainty of evidence for each outcome. Evidence from non-randomised studies will initially be rated as low but may be upgraded or downgraded based on risk of bias, inconsistency, indirectness, imprecision and publication bias.

## Ethics and dissemination

No primary data will be collected; therefore, ethical approval is not required. The findings will be disseminated via publication in a peer-reviewed journal and presentations at thoracic surgery and respiratory medicine conferences.

## PROSPERO registration

This protocol will be registered prospectively on the International Prospective Register of Systematic Reviews (PROSPERO). The PROSPERO registration number will be provided once issued.

## Timeline

Protocol development and registration: May 2026.

Literature search and screening: May 2026.

Data extraction and risk-of-bias assessment: May-June 2026.

Data analysis and drafting of manuscript: June-July 2026.

Manuscript submission: August 2026.

## Authors' contributions

Munir Adamu Bala conceived the review question and drafted the protocol. Ahmad Asqalan and Vasileios Kouritas provided methodological input and critically revised the protocol. All authors approved the final manuscript.

## Conflicts of interest

The authors declare no conflicts of interest.

## Funding

None
