# Supplementary material for: Outcomes of combined lung cancer resection and lung volume reduction surgery in adults with severe emphysema; a systematic review and meta-analysis
Source: Front Oncol. 2026 Aug 19;16:1916431. doi: 10.3389/fonc.2026.1916431 (PMC13533703; doi:10.3389/fonc.2026.1916431)
Supplement: Supplementary file 2 [file DataSheet2.pdf]

NIHR | National Institute for Health and Care Research

PROSPERO
International prospective register of systematic reviews

Outcomes of Combined Lung Cancer Resection and Lung Volume Reduction Surgery in Adults with Severe Emphysema: A Systematic Review and Meta-analysis

Munir Adamu Bala, Vasileios Kouritas, Ahmad Asqalan, Beshoy Effat Elkomos, Ayush Jha, Ra'fat Tawalbeh, Mohammad Tayeh, Jameel Ismail Ahmad

Citation
Munir Adamu Bala, Vasileios Kouritas, Ahmad Asqalan, Beshoy Effat Elkomos, Ayush Jha, Ra'fat Tawalbeh, Mohammad Tayeh, Jameel Ismail Ahmad. Outcomes of Combined Lung Cancer Resection and Lung Volume Reduction Surgery in Adults with Severe Emphysema: A Systematic Review and Meta-analysis. PROSPERO 2026 CRD420261397321. Available from https://www.crd.york.ac.uk/PROSPERO/view/CRD420261397321.

REVIEW TITLE AND BASIC DETAILS

Review title
Outcomes of Combined Lung Cancer Resection and Lung Volume Reduction Surgery in Adults with Severe Emphysema: A Systematic Review and Meta-analysis

Review type
None

Condition or domain being studied
Lung cancer; Pulmonary Emphysema; Chronic Obstructive Pulmonary Disease

Rationale for the review
Lung cancer frequently coexists with advanced emphysema or chronic obstructive

pulmonary disease (COPD). Patients with severe emphysema often have limited pulmonary reserve, and this may preclude definitive lung-cancer resection. Lung volume reduction surgery (LVRS) has been shown to improve symptoms and lung function in well-selected emphysema patients. Combining LVRS with lung cancer resection is therefore hypothesised to allow complete oncologic resection while providing a "lung-volume-reduction effect"; removal of hyper-expanded, non-functional lung; that stabilises or improves postoperative pulmonary function. Early case series report that lobectomy or wedge resection supplemented with LVRS can achieve acceptable morbidity and survival. However, the evidence base remains sparse and heterogeneous, and there is no consolidated synthesis of perioperative, functional and oncologic outcomes compared with standard procedures.

Review objectives

To evaluate perioperative outcomes (30-day or in-hospital mortality and major complications) in adults with severe emphysema undergoing lung cancer resection combined with LVRS.

Keywords

Lung volume reduction; Lung cancer surgery; Chronic obstructive pulmonary disease COPD; Lung cancer

Country

United Kingdom

ELIGIBILITY CRITERIA

Population

Included

Adults (≥18 years) with radiologically or histologically confirmed severe emphysema (with or without COPD) and primary lung cancer. Studies including mixed severity are eligible if data for severe emphysema can be extracted.

Intervention(s) or exposure(s)

Included

Lung cancer resection (lobectomy, segmentectomy, wedge resection or pneumonectomy) performed simultaneously with LVRS, including formal LVRS, LVRS-like resection (lobectomy acting as LVRS)

Comparator(s) or control(s)

Included

Lung cancer resection alone in patients with emphysema,

LVRS alone,

Staged LVRS and cancer resection,

No comparator (single-arm studies).

Study design
Both randomized and nonrandomized study types will be included.

Included
Randomised controlled trials, prospective or retrospective cohort studies, case–control studies and case series with ≥5 patients. Conference abstracts will be included if sufficient data are available.

Excluded
Case reports with <5 patients will be narratively summarised but excluded from quantitative synthesis.

Context
A study should be considered eligible if it reports a planned strategy in which lung cancer

resection is performed with an explicit volume-reduction intent or component in

emphysematous patients. That includes simultaneous or staged surgical LVRS done with

wedge resection, segmentectomy, lobectomy, or another oncologic resection.

TIMELINE OF THE REVIEW

Date of first submission to PROSPERO
28 May 2026

Review timeline
Review start date: 16 May 2026. Review end date: 31 August 2026.

Date of registration in PROSPERO
31 May 2026

AVAILABILITY OF FULL PROTOCOL

Availability of full protocol
A full protocol has been written and uploaded to PROSPERO. The protocol may be

accessed through this link
https://www.crd.york.ac.uk/PROSPEROFILES/1435109ec2549a15ac65be2b71c75cf3.pdf.

SEARCHING AND SCREENING

Search for unpublished studies
Only published studies will be sought.

Main sources that will be searched
The main sources to be searched are *CENTRAL - Cochrane Central Register of Controlled Trials*, *Clinicaltrials.gov*, *Embase*, *Google Scholar*, *MEDLINE*, *PubMed* and *Scopus*.

Other sources that will be searched
Semantic Scholar

Open Alex

Search language restrictions
There are no language restrictions.

Search date restrictions
There are no search date restrictions.

Other methods of identifying studies
Other studies will be identified by: *looking through all the articles that cite the papers included in the review ("snowballing" or forward citation searching)*, *reference list checking (backward citation searching)* and *searching trial or study registers*.

Link to search strategy
A full search strategy is available in the full protocol as described in the *Availability of full protocol* section

Selection process
Studies will be screened independently by at least two people (or person/machine combination) with a process to resolve differences.

Other relevant information about searching and screening
None

DATA COLLECTION PROCESS

Data extraction from published articles and reports
Data will be extracted independently by at least two people (or person/machine

combination) with a process to resolve differences.

Authors will be asked to provide any required data not available in published reports.

Study risk of bias or quality assessment

Risk of bias will be assessed using: *Cochrane RoB-2*, *JBI tools* and *ROBINS-I*

Data will be assessed independently by at least two people (or person/machine combination) with a process to resolve differences.

Additional information will be sought from study investigators if required information is unclear or unavailable in the study publications/reports.

Reporting bias assessment

Risk of bias due to missing results will not be assessed

Certainty assessment

Certainty of findings will not be assessed

OUTCOMES TO BE ANALYSED

Main outcomes

perioperative mortality (≤30 days/in-hospital) and major postoperative complications (e.g., prolonged air leak, respiratory failure, reintubation, atrial fibrillation, pneumothorax).

Additional outcomes

changes in FEV_1 and FEV_1% predicted, DLCO, residual volume, exercise capacity (6-minute walk test), dyspnoea scores; length of hospital/ICU stay; oncologic outcomes (overall survival, disease-free survival, recurrence).

PLANNED DATA SYNTHESIS

Strategy for data synthesis

Quantitative synthesis will be performed if ≥3 studies with comparable design and outcome measures are available.

Effect measures

• Dichotomous outcomes (mortality, complications) will be pooled as risk ratios (RR) or odds ratios (OR) with 95% confidence intervals (CI). Single-arm proportions will be pooled using logit transformation.

• Continuous outcomes (FEV_1, DLCO, etc.) will be pooled as mean differences (MD) when measured uniformly; otherwise standardised mean differences (SMD) will be

used.

• Time-to-event outcomes (overall and disease-free survival) will be summarised as hazard ratios (HR) when reported; otherwise survival proportions at common time points will be extracted.

Model selection;

A random-effects model (DerSimonian–Laird or Restricted Maximum Likelihood) will be applied to account for heterogeneity among studies. Fixed-effect models will be used in sensitivity analyses.

Heterogeneity assessment;

Statistical heterogeneity will be assessed using the I^2 statistic (values of 25%, 50% and 75% representing low, moderate and high heterogeneity) and the χ^2 test (p < 0.10 considered significant). Sources of heterogeneity will be explored through subgroup and sensitivity analyses (see below)

CURRENT REVIEW STAGE

Stage of the review at this submission

Review stage	Started	Completed
Pilot work		
Formal searching/study identification		
Screening search results against inclusion criteria		
Data extraction or receipt of IPD		
Risk of bias/quality assessment		
Data synthesis		

Review status

The review is currently planned or ongoing.

Publication of review results

Results of the review will be published in English.

REVIEW AFFILIATION, FUNDING AND PEER REVIEW

Review team members

Dr Munir Adamu Bala (review guarantor). ORCID: 0009-0008-2657-3364. Norfolk and Norwich Universty Hospital. England.

No conflict of interest declared.

Mr Vasileios Kouritas. Norfolk and Norwich University Hospitals NHS Foundation Trust. England.

No conflict of interest declared.

Dr Ahmad Asqalan. ORCID: 0000-0003-2388-8025. Norfolk and Norwich University Hospitals NHS Foundation Trust. England.

No conflict of interest declared.

Dr Beshoy Effat Elkomos. ORCID: 0000-0001-6663-4266. Royal United Hospital: Bath, England, GB. England.

No conflict of interest declared.

Dr Ayush Jha. ORCID: 0009-0000-7585-5992. Norfolk and Norwich University Hospital: Norwich, England, GB. England.

No conflict of interest declared.

Dr Ra'fat Tawalbeh. ORCID: 0000-0002-4075-2683. Norfolk and Norwich University Hospital. England.

No conflict of interest declared.

Mohammad Tayeh. ORCID: 0009-0000-0495-6622. Faculty of Medicine, Jordan University of Science and Technology. Jordan.

No conflict of interest declared.

Professor Jameel Ismail Ahmad. ORCID: 0000-0001-9112-8445. Aminu Kano Teaching Hospital, Kano. Nigeria.

No conflict of interest declared.

Named contact

Mr Vasileios Kouritas (Vasileios.Kouritas@nnuh.nhs.uk). Norfolk and Norwich University Hospitals NHS Foundation Trust. England.

Review affiliation

Norfolk and Norwich University Hospital

Funding source

Review has no funding and no agreed support from an academic institution and is done in authors’ own time.

Peer review
There has been no peer review of this planned review.

ADDITIONAL INFORMATION

Review conflict of interest
Declared individual interests are recorded under team member details.. No additional interests are recorded for this review.

Medical Subject Headings
Emphysema; Lung Neoplasms; Pneumonectomy; Pulmonary Disease, Chronic Obstructive

SIMILAR REVIEWS

Check for similar records already in PROSPERO
PROSPERO identified a number of existing PROSPERO records that were similar to this one (last check made on 22 May 2026). These are shown below along with the reasons given by that the review team for the reviews being different and/or proceeding.

- Safety and efficacy of noninvasive ventilation in lung volume reduction surgery (LVRS) with thoracoscopy [published 3 December 2023] [CRD42023478995]. The review was judged **not to be similar**
- Extrapulmonary effects of lung volume reduction treatment in patients with severe emphysema: a systematic review [published 30 December 2023] [CRD42023495135]. The review was judged **not to be similar**
- Surgical Resection Extent in Stage IA Non-Small Cell Lung Cancer: umbrella review of evidence [published 28 July 2025] [CRD420251082817]. The review was judged **not to be similar**

PROSPERO version history

- Version 1.0, published 31 May 2026

Disclaimer
The content of this record displays the information provided by the review team. PROSPERO does not peer review registration records or endorse their content.

PROSPERO accepts and posts the information provided in good faith; responsibility for record content rests with the review team. The guarantor for this record has

affirmed that the information provided is truthful and that they understand that deliberate provision of inaccurate information may be construed as scientific misconduct.

PROSPERO does not accept any liability for the content provided in this record or for its use. Readers use the information provided in this record at their own risk.

Any enquiries about the record should be referred to the named review contact
