# Supplementary material for: Outcomes of combined lung cancer resection and lung volume reduction surgery in adults with severe emphysema; a systematic review and meta-analysis
Source: Front Oncol. 2026 Aug 19;16:1916431. doi: 10.3389/fonc.2026.1916431 (PMC13533703; doi:10.3389/fonc.2026.1916431)
Supplement: Supplementary file 3 [file Table2.docx]

**Supplementary File 4**

Complete database-specific search strategies — search date: 27 May 2026

The searches combined four concepts: emphysema/COPD; lung volume reduction; lung neoplasm; and cancer resection performed simultaneously with or in a volume-reduction concept. No language or publication-date limits were applied. Reference-list and forward-citation checking supplemented the electronic searches.

PubMed

(("Pulmonary Emphysema"[Mesh] OR emphysema[tiab] OR "severe emphysema"[tiab] OR "Chronic Obstructive Pulmonary Disease"[Mesh] OR COPD[tiab]) AND ("Lung Volume Reduction Surgery"[Mesh] OR "lung volume reduction"[tiab] OR LVRS[tiab] OR "volume reduction effect"[tiab] OR "lobar volume reduction"[tiab]) AND ("Lung Neoplasms"[Mesh] OR "Carcinoma, Non-Small-Cell Lung"[Mesh] OR "lung cancer"[tiab] OR NSCLC[tiab] OR "pulmonary nodule"[tiab]) AND (resection[tiab] OR lobectomy[tiab] OR segmentectomy[tiab] OR "wedge resection"[tiab] OR surgery[tiab]) AND (combined[tiab] OR concomitant[tiab] OR concurrent[tiab] OR simultaneous[tiab] OR synchronous[tiab]))

Scopus

TITLE-ABS-KEY((emphysema OR COPD) AND ("lung volume reduction" OR LVRS OR "volume reduction effect" OR "lobar volume reduction") AND ("lung cancer" OR NSCLC OR "pulmonary nodule") AND (resection OR lobectomy OR segmentectomy OR "wedge resection" OR surgery) AND (combined OR concomitant OR concurrent OR simultaneous OR synchronous))

Web of Science Core Collection

TS=((emphysema OR COPD) AND ("lung volume reduction" OR LVRS OR "volume reduction effect" OR "lobar volume reduction") AND ("lung cancer" OR NSCLC OR "pulmonary nodule") AND (resection OR lobectomy OR segmentectomy OR "wedge resection" OR surgery) AND (combined OR concomitant OR concurrent OR simultaneous OR synchronous))

CENTRAL

([mh "Pulmonary Emphysema"] OR [mh "Chronic Obstructive Pulmonary Disease"] OR emphysema:ti,ab,kw OR COPD:ti,ab,kw) AND ([mh "Lung Volume Reduction Surgery"] OR "lung volume reduction":ti,ab,kw OR LVRS:ti,ab,kw OR "volume reduction effect":ti,ab,kw) AND ([mh "Lung Neoplasms"] OR [mh "Carcinoma, Non-Small-Cell Lung"] OR "lung cancer":ti,ab,kw OR NSCLC:ti,ab,kw) AND (resection:ti,ab,kw OR lobectomy:ti,ab,kw OR segmentectomy:ti,ab,kw OR "wedge resection":ti,ab,kw) AND (combined:ti,ab,kw OR concomitant:ti,ab,kw OR simultaneous:ti,ab,kw)

Semantic Scholar

Query variants searched: (1) "lung cancer" "lung volume reduction surgery"; (2) NSCLC LVRS lobectomy emphysema; (3) "volume reduction effect" lung cancer resection; (4) simultaneous cancer resection LVRS emphysema. Results were screened for the complete eligibility criteria rather than relying on platform relevance ranking.

OpenAlex

Keyword query variants searched in title/abstract/full-text search: (1) "lung cancer" AND "lung volume reduction"; (2) NSCLC AND LVRS; (3) emphysema AND lobectomy AND "volume reduction effect"; (4) pulmonary nodule AND simultaneous LVRS. Works were deduplicated by DOI/title against the master library.

ClinicalTrials.gov

Condition/disease: lung cancer OR pulmonary neoplasm; Other terms: (lung volume reduction OR LVRS) AND (combined OR simultaneous OR concomitant); Study type: all; Status: all; First posted: no restriction.

WHO ICTRP

Title/intervention/condition search variants: "lung volume reduction" AND "lung cancer"; LVRS AND pulmonary neoplasm; simultaneous LVRS resection; emphysema lobectomy volume reduction. Recruitment status, country, and registration date were unrestricted.

Citation searching

Backward reference checking was performed for every included report and relevant review. Forward citation searching used the included reports as seed records. Potentially overlapping institutional reports were compared by centre, recruitment period, sample size, procedures, and outcome reporting.
