# Supplementary material for: Outcomes of combined lung cancer resection and lung volume reduction surgery in adults with severe emphysema; a systematic review and meta-analysis
Source: Front Oncol. 2026 Aug 19;16:1916431. doi: 10.3389/fonc.2026.1916431 (PMC13533703; doi:10.3389/fonc.2026.1916431)
Supplement: Supplementary file 4 [file Table3.docx]

**Data Synthesis Decision Log**

**1. Executive summary of synthesis decisions**

| Decision area | Summary |
| --- | --- |
| Primary quantitative approach | Single-arm meta-analysis was used for outcomes where at least three comparable studies reported extractable data. Comparative meta-analysis was not performed because fewer than three comparable comparative datasets were available. |
| Primary model | Random-effects models were used because the included studies differed in surgical era, patient selection, emphysema distribution, tumour stage, and operative approach. |
| Proportions | Mortality, air leak and recurrence were synthesised as logit-transformed single-arm proportions with continuity correction for zero-event studies. |
| Continuous outcomes | FEV1 change was pooled as mean difference in litres. Where SD of change was not reported, it was estimated from preoperative and postoperative SDs using an assumed within-person correlation. |
| Survival | Overall survival was kept descriptive because hazard ratios, standard errors and numbers at risk were not consistently reported. |
| Publication bias | Funnel plots and Egger tests were not performed because no meta-analysis contained at least ten studies. |
| Hayashi 1999 | Included in the review and sensitivity discussion, but not in the primary mortality, prolonged-air-leak or FEV1-litre meta-analyses because its outcomes did not match the strict primary outcome definitions or the main measurement scale. |

**2. Protocol rules that governed synthesis**

The following protocol-defined rules were applied before any outcome was pooled:

Quantitative synthesis was only undertaken when at least three studies had comparable design and comparable outcome reporting.

Dichotomous outcomes were pooled as risk ratios or odds ratios for comparative datasets where appropriate; single-arm proportions were pooled using a logit transformation.

Continuous outcomes were pooled as mean differences when studies used the same measurement unit, or as standardised mean differences if measurement scales differed.

Time-to-event outcomes were planned as hazard ratios where available; otherwise survival proportions at common time points were extracted.

A random-effects model was used as the primary model to account for clinical and methodological heterogeneity.

Risk of bias was considered when interpreting results and when defining sensitivity analyses.

Publication bias assessment was reserved for analyses with at least ten studies.

**3. General study-level decision principles**

The synthesis did not treat every eligible article as automatically poolable. A study could be included in the review but excluded from a specific meta-analysis if it did not report that outcome in a compatible form. The following principles were applied:

| Principle | How it was applied |
| --- | --- |
| Avoid double counting | When two reports appeared to describe overlapping cohorts from the same institution, the most complete or latest dataset was preferred for primary quantitative synthesis. Earlier or educational reports were retained narratively if useful. |
| Cancer-only focus | Studies dominated by benign pulmonary nodules were not included in primary cancer-specific pooled outcomes unless cancer-only data were extractable. |
| Endpoint compatibility | The time point and definition of an outcome had to match the primary analysis. For example, a death reported at 2 months as part of prognosis was not treated as 30-day/in-hospital mortality. |
| Measurement-scale compatibility | FEV1 in litres was pooled separately from FEV1 percent predicted. Mixing litres and percentages in one mean-difference model was avoided. |
| Risk-of-bias sensitivity | Studies with serious or high risk of bias were either excluded from low-risk sensitivity analyses or used only where the outcome was objective and otherwise extractable. |
| No causal inference from non-causal comparisons | Risk-stratified groups or historically dissimilar controls were not treated as causal intervention comparisons. |

**4. Study-level inclusion and pooling decisions**

The table below records the main study-level decisions used for the primary and sensitivity synthesis. “Included” means included in at least one primary quantitative analysis; “narrative/sensitivity” means included in the systematic review but not the primary pooled estimate for one or more outcomes.

| Study/report | Synthesis decision | Reasoning |
| --- | --- | --- |
| Choong 2004 | Included | Updated Barnes/Washington University cohort with clear denominator, hospital mortality, prolonged air leak, FEV1 follow-up and survival estimates. Used in mortality, prolonged air leak, FEV1 and descriptive survival. |
| DeMeester 1998 | Narrative / superseded | Early five-patient report from the same institutional programme later represented within the larger Choong 2004 cohort. Excluded from primary pooling to avoid double counting. |
| McKenna 1996 | Included | Distinct LVRS programme with clinical stage I lung cancers; clear denominator, perioperative mortality and prolonged air leak. Included in mortality and air leak analyses. |
| Ojo 1997 | Narrative / safety only | Mostly pulmonary nodules rather than cancer-only cohort; only three neoplastic lesions. The control group underwent standard lobectomy and was not comparable to combined LVRS-cancer patients. Serious confounding meant comparative pooling would be misleading. |
| DeRose 1998 | Included | Columbia-Presbyterian series with explicit inclusion and exclusion criteria, clear denominator and extractable postoperative death, prolonged air leak and FEV1 data. Preferred over Allen/DeRose educational overlap. |
| Allen/DeRose 1997 | Narrative / superseded | Educational AORN report overlaps with the Columbia-Presbyterian experience; DeRose 1998 is the peer-reviewed clinical report with clearer outcome extraction. Excluded from pooling to avoid double counting. |
| Hayashi 1999 | Review + sensitivity/narrative | Eligible case series of lung cancer with severe emphysema, but the reported outcomes were not compatible with the primary pooled endpoints. Detailed reasoning is provided in Section 6. |
| Ambrogi 2000 | Narrative / superseded | Early Rome/Tor Vergata experience likely overlaps with the later Pompeo 2003 quality-of-life cohort. Pompeo 2003 was preferred for primary pooling because it is larger and provides more complete functional and follow-up data. |
| Pompeo 2003 | Included | More comprehensive Rome/Tor Vergata cohort with stage I NSCLC, clear hospital mortality, prolonged air leak and FEV1 change. Included in mortality, air leak, FEV1 and descriptive survival. |
| Teschner 2003 | Narrative / excluded from primary pooling | Small case series with high risk of bias and limited outcome reporting. Not used in primary pooled estimates to avoid unstable and poorly defined contributions. |
| Edwards 2001 | Included selectively | Lobectomy with volume-reduction effect in emphysematous target lobes. Included for objective single-arm/group-specific mortality where extractable. Comparative inference between ppoFEV1 strata was not pooled because groups were risk strata, not randomised interventions. |
| Tang 2009 | Included selectively | Randomised clinical study but mixed pulmonary and oesophageal neoplasms. Only pulmonary-neoplasm subgroup data were used where extractable. Oesophageal subgroup and mixed overall data were not pooled with lung cancer outcomes. |
| Caviezel 2016 abstract | Superseded | Conference abstract from the Zurich group. Excluded from primary pooling because the 2018 full paper provides a larger, more complete and peer-reviewed dataset. |
| Caviezel 2018 | Included | Full Zurich series with clear 90-day mortality, prolonged air leak, FEV1 change and survival data. Used instead of the abstract. |

**5. Outcome-level synthesis decisions**

**5.1 Perioperative mortality**

Primary pooled estimate: 7 studies, 113 patients, 3 deaths; pooled estimate 7.3% (95% CI 3.4–15.1). Low-risk-of-bias sensitivity: 5 studies, 76 patients, 1 death; pooled estimate 5.1% (95% CI 1.8–13.6).

| Decision | Reasoning |
| --- | --- |
| Included studies | Choong 2004, McKenna 1996, Caviezel 2018, DeRose 1998, Edwards 2001, Pompeo 2003 and Tang 2009 pulmonary-neoplasm subgroup. |
| Why single-arm pooling? | Most studies were uncontrolled case series. Comparable comparative datasets were insufficient, so event proportions were more appropriate than risk ratios. |
| Why logit transformation? | Mortality events were sparse and several studies had zero deaths. Logit-transformed proportions with continuity correction allow zero-event studies to contribute while keeping estimates within 0–100%. |
| Why random effects? | The studies differed by era, extent of resection, LVRS strategy, surgical approach and baseline emphysema severity. Random effects better reflects this heterogeneity than assuming one common true effect. |
| Why include Edwards 2001? | Although ROBINS-I was serious for causal comparison, group-specific 30-day mortality in the ppoFEV1<40 group was objective and extractable. It was therefore used as a single-arm estimate, not as a causal comparison. |
| Why include Tang 2009 cautiously? | Tang reported no surgical deaths and included a pulmonary-neoplasm LVRS subgroup. Only the pulmonary-neoplasm subgroup denominator was used for lung-cancer-relevant synthesis; mixed oesophageal data were not pooled. |
| Why exclude Hayashi 1999 from primary mortality? | Deaths were reported at 2 months and 17 months, not as 30-day or in-hospital mortality. The 2-month death was therefore used only in broader early-mortality sensitivity analysis. |

**5.2 Prolonged air leak**

Primary pooled estimate: 5 studies, 76 patients, 28 events; pooled estimate 38.0% (95% CI 26.9–50.6). Broader any/unspecified air-leak sensitivity: 7 studies, 128 patients, 44 events; pooled estimate 35.7% (95% CI 27.9–44.3).

| Decision | Reasoning |
| --- | --- |
| Primary definition | Only studies explicitly reporting prolonged air leak or a clearly comparable postoperative prolonged air leak event were included in the primary analysis. |
| Included studies | Choong 2004, McKenna 1996, Caviezel 2018, DeRose 1998 and Pompeo 2003. |
| Why not pool all air leak reports together? | Studies used different thresholds and terminology. Pooling “prolonged air leak” with vague “bronchial air leakage” would reduce clinical interpretability. |
| Why sensitivity analysis? | Because air leak is clinically important and some studies reported less strictly defined air-leak outcomes. The sensitivity analysis tested whether including broader definitions materially changed the estimate. |
| Why exclude Hayashi 1999 from primary air leak? | Hayashi reported bronchial air leakage requiring postoperative treatment, but the available reporting did not provide a comparable prolonged-air-leak threshold such as >7 days or >14 days. |
| Clinical interpretation | Air leak is expected to be common in emphysematous parenchyma and in procedures involving stapled volume reduction. The pooled estimate should be interpreted as a morbidity signal, not a precise universal risk. |

**5.3 FEV1 change**

Exploratory pooled estimate: 4 studies, 74 patients; mean difference +0.26 L (95% CI +0.20 to +0.32).

| Decision | Reasoning |
| --- | --- |
| Measurement scale | The primary FEV1 synthesis used litres, not percent predicted, because mean difference requires the same unit of measurement. |
| Included studies | Choong 2004, DeRose 1998, Pompeo 2003 and Tang 2009 pulmonary-neoplasm subgroup. |
| Why exploratory? | The analysis is based on pre-post changes from observational cohorts, not randomised comparisons. The estimate may be affected by survivorship and selective follow-up testing. |
| SD_change estimation | Where studies did not report the SD of the paired change, SD_change was estimated from preoperative and postoperative SDs using an assumed within-person correlation of r=0.5. Sensitivity analyses used r=0.25 and r=0.75. |
| Why not include studies reporting only FEV1 percent predicted? | Combining litres and percent predicted in a single mean-difference model would be inappropriate. Percent-predicted values can be summarised narratively or analysed separately if at least three compatible datasets are available. |
| Why exclude Hayashi 1999 from primary FEV1 litres? | Hayashi’s main extracted postoperative function data were reported as FEV1.0%/percent values and individual or summary percent-based results rather than a directly compatible mean change in litres with SD_change. Including it would require additional derivation and would mix measurement scales. |
| Interpretation | The direction of change supports the physiologic rationale that resecting hyperinflated, poorly functioning lung may offset expected functional loss after cancer resection. |

**5.4 Recurrence and oncologic outcomes**

Reported recurrence was pooled cautiously where extractable: 4 studies, 58 patients, 12 events; pooled estimate 25.1% (95% CI 15.4–38.2). Overall survival was not formally pooled.

| Decision | Reasoning |
| --- | --- |
| Recurrence pooling | A cautious pooled proportion was produced only for studies that reported recurrence events and denominator. It was not treated as disease-free survival. |
| Why “with caution”? | Follow-up duration, staging procedures, lymph node assessment and definitions of recurrence varied substantially. |
| Why not pool overall survival? | Studies reported survival as Kaplan-Meier estimates or actuarial survival at different time points, generally without hazard ratios, standard errors or number-at-risk tables. |
| Descriptive survival decision | Survival was summarised narratively using reported 1-, 3- and 5-year estimates where available. This avoids creating false precision from incompatible data. |
| Oncologic interpretation | The data show feasibility of resection in high-risk patients but do not establish oncologic equivalence to standard lobectomy, sublobar resection or stereotactic radiotherapy. |

**5.5 Comparative outcomes**

| Decision | Reasoning |
| --- | --- |
| Why comparative meta-analysis was not performed | There were fewer than three comparable comparative datasets for any outcome. Available comparisons differed in design, population and purpose. |
| Tang 2009 | The study included pulmonary and oesophageal neoplasms and the reporting did not provide a clean set of lung-cancer-only comparative outcomes for all endpoints. Therefore it was summarised, and only compatible pulmonary-neoplasm data were used selectively. |
| Edwards 2001 | The comparison was between ppoFEV1 risk strata rather than between randomized treatment strategies. It was not interpreted as causal evidence that one intervention is superior. |
| Ojo 1997 | Controls underwent standard lobectomy and had much better baseline lung function. The study group included mostly benign nodules. Comparative pooling would have been confounded and indirect. |

**6. Specific reasoning for Hayashi 1999**

Hayashi 1999 was not omitted from the systematic review. It was retained as an eligible case series and considered in sensitivity/narrative synthesis. The key issue is that eligibility for the review is not the same as eligibility for every meta-analysis.

| Issue | Decision and reasoning |
| --- | --- |
| Review eligibility | The study included patients with lung cancer complicated by severe pulmonary emphysema who underwent lung reduction/resection surgery. It therefore fits the clinical question and should remain in the review. |
| Risk of bias | The study was judged moderate risk of bias because it was a small uncontrolled case series with mixed stages and incomplete reporting for some outcome measures. This does not automatically exclude it, but it lowers certainty. |
| Mortality decision | The reported deaths occurred at 2 months and 17 months. The primary mortality outcome in the protocol is 30-day or in-hospital mortality. The 2-month death cannot be confidently classified as 30-day or in-hospital mortality, and the 17-month death is clearly late follow-up. Therefore, Hayashi was excluded from the primary mortality pool. |
| Mortality sensitivity | The 2-month death was included in a broader early-mortality sensitivity analysis to test whether its inclusion changed the overall estimate. This is transparent and avoids discarding relevant safety information. |
| Air leak decision | The study reports bronchial air leakage requiring postoperative treatment, but not a clearly comparable definition of prolonged air leak such as >7 days or >14 days. It was therefore excluded from the primary prolonged-air-leak analysis. It may be discussed under broader postoperative air-leak morbidity. |
| FEV1 decision | The primary FEV1 meta-analysis pooled mean change in litres. Hayashi reports postoperative lung function in a form that is not directly compatible with the litre-based model, mainly FEV1.0%/percent values and incomplete SD_change information. It was therefore not included in the primary FEV1-litre meta-analysis. |
| Oncologic outcomes | Survival and recurrence are reported descriptively with short and variable follow-up. These data are best summarised narratively rather than pooled with studies providing actuarial or Kaplan-Meier estimates. |
| How to describe in manuscript | Hayashi 1999 should be described as included in the systematic review but excluded from primary quantitative syntheses because reported endpoints did not match the primary outcome definitions. The 2-month death can be noted in a sensitivity analysis. |

**7. Sensitivity analyses and why they were used**

| Sensitivity analysis | Reasoning and interpretation |
| --- | --- |
| Low-risk-of-bias mortality analysis | Used to test whether mortality estimates were driven by studies with serious/high risk of bias. Result remained low but imprecise: 5.1% (95% CI 1.8–13.6). |
| Mortality including Hayashi 1999 2-month death | Used to test a broader early-mortality definition. Result: 8.2% (95% CI 4.1–15.7), similar to the primary estimate. |
| Any/unspecified air leak | Used to test whether including broader air-leak definitions materially changed the estimate. Result: 35.7% (95% CI 27.9–44.3), close to the primary prolonged-air-leak estimate. |
| FEV1 correlation assumptions | Used because SD_change had to be estimated for several studies. Results were stable across assumed correlations r=0.25, r=0.50 and r=0.75. |

**8. Reporting bias and certainty-of-evidence decisions**

| Area | Decision and reasoning |
| --- | --- |
| Publication bias | Not assessed using funnel plots or Egger regression because no pooled analysis included ten or more studies. With fewer studies, these tests are underpowered and potentially misleading. |
| GRADE starting point | Most evidence came from observational case series or non-randomised studies; therefore certainty started low and was commonly downgraded. |
| Downgrade: risk of bias | Selection bias was substantial because patients were highly selected for favourable anatomy, resectability and ability to tolerate surgery. |
| Downgrade: inconsistency/indirectness | Studies differed in surgical approach, tumour stage, emphysema pattern, outcome definitions and era of care. |
| Downgrade: imprecision | Sample sizes and event counts were small, and confidence intervals were wide for key outcomes. |
| Overall certainty | Very low for the key pooled outcomes. Findings should be interpreted as feasibility evidence rather than definitive comparative effectiveness evidence. |
