# Supplementary material for: Outcomes of combined lung cancer resection and lung volume reduction surgery in adults with severe emphysema; a systematic review and meta-analysis
Source: Front Oncol. 2026 Aug 19;16:1916431. doi: 10.3389/fonc.2026.1916431 (PMC13533703; doi:10.3389/fonc.2026.1916431)
Supplement: Supplementary file 5 [file Table4.docx]

The input values below are transcribed from the review's extraction workbook.

The script deliberately separates observed study proportions from the

continuity-corrected values used in the random-effects logit model.

"""

from __future__ import annotations

import json

import math

import os

from pathlib import Path

os.environ.setdefault("MPLCONFIGDIR", "/tmp/mplconfig-lvrs-review")

import matplotlib.pyplot as plt

import numpy as np

from matplotlib.patches import Circle, Ellipse, FancyArrowPatch, FancyBboxPatch, Polygon

from scipy.stats import norm

OUT = Path(__file__).resolve().parent / "deliverables" / "figures"

OUT.mkdir(parents=True, exist_ok=True)

NAVY = "#0B2D4D"

BLUE = "#2F6FA3"

TEAL = "#167C80"

ORANGE = "#E67E22"

RED = "#B23A48"

GREEN = "#2D7A4B"

GREY = "#58636F"

LIGHT = "#EEF4F8"

PALE_BLUE = "#DCEAF4"

PALE_ORANGE = "#FCEBD8"

plt.rcParams.update(

{

"font.family": "DejaVu Sans",

"font.size": 9,

"axes.titlesize": 12,

"axes.labelsize": 9,

"figure.dpi": 150,

"savefig.bbox": "tight",

"svg.fonttype": "none",

}

)

MORTALITY = [

("Choong 2004", 0, 21),

("McKenna 1996", 0, 11),

("Caviezel 2018", 0, 14),

("DeRose 1998*", 1, 14),

("Edwards 2001", 2, 14),

("Pompeo 2003", 0, 16),

("Tang 2009*", 0, 23),

]

PAL = [

("Choong 2004", 11, 21),

("McKenna 1996", 5, 11),

("Caviezel 2018", 3, 14),

("DeRose 1998*", 3, 14),

("Pompeo 2003", 6, 16),

]

RECURRENCE = [

("Choong 2004", 5, 21),

("McKenna 1996", 0, 11),

("Hayashi 1999", 3, 10),

("Pompeo 2003", 4, 16),

]

FEV1 = [

("Choong 2004", 0.30, 0.0577350269),

("DeRose 1998*", 0.21, 0.1271652468),

("Pompeo 2003", 0.28, 0.05),

("Tang 2009*", 0.18, 0.0642007720),

]

def inv_logit(value: float | np.ndarray) -> float | np.ndarray:

return 1.0 / (1.0 + np.exp(-value))

def wilson_ci(events: int, total: int, alpha: float = 0.05) -> tuple[float, float]:

"""Wilson score confidence interval for a binomial proportion."""

z = norm.ppf(1 - alpha / 2)

p = events / total

den = 1 + z * z / total

centre = (p + z * z / (2 * total)) / den

half = z * math.sqrt(p * (1 - p) / total + z * z / (4 * total * total)) / den

return max(0.0, centre - half), min(1.0, centre + half)

def dl_logit_proportion(rows: list[tuple[str, int, int]], correction: float = 0.5) -> dict:

"""DerSimonian-Laird random-effects meta-analysis of logit proportions.

A correction of 0.5 is added to both event and non-event cells for every

study because the analysis contains zero-event studies.

"""

x = np.array([r[1] for r in rows], dtype=float)

n = np.array([r[2] for r in rows], dtype=float)

p = (x + correction) / (n + 2 * correction)

yi = np.log(p / (1 - p))

vi = 1 / (x + correction) + 1 / (n - x + correction)

wi = 1 / vi

fixed = np.sum(wi * yi) / np.sum(wi)

q = float(np.sum(wi * (yi - fixed) ** 2))

df = len(rows) - 1

c_value = float(np.sum(wi) - np.sum(wi**2) / np.sum(wi))

tau2 = max(0.0, (q - df) / c_value) if c_value > 0 else 0.0

weights = 1 / (vi + tau2)

pooled = float(np.sum(weights * yi) / np.sum(weights))

se = math.sqrt(1 / float(np.sum(weights)))

lower, upper = pooled - 1.96 * se, pooled + 1.96 * se

i2 = max(0.0, (q - df) / q * 100) if q > 0 else 0.0

return {

"events": int(np.sum(x)),

"participants": int(np.sum(n)),

"crude": float(np.sum(x) / np.sum(n)),

"estimate": float(inv_logit(pooled)),

"lower": float(inv_logit(lower)),

"upper": float(inv_logit(upper)),

"tau2": tau2,

"q": q,

"i2": i2,

}

def dl_mean_difference(rows: list[tuple[str, float, float]]) -> dict:

values = np.array([r[1] for r in rows], dtype=float)

variances = np.array([r[2] ** 2 for r in rows], dtype=float)

wi = 1 / variances

fixed = np.sum(wi * values) / np.sum(wi)

q = float(np.sum(wi * (values - fixed) ** 2))

df = len(rows) - 1

c_value = float(np.sum(wi) - np.sum(wi**2) / np.sum(wi))

tau2 = max(0.0, (q - df) / c_value) if c_value > 0 else 0.0

weights = 1 / (variances + tau2)

pooled = float(np.sum(weights * values) / np.sum(weights))

se = math.sqrt(1 / float(np.sum(weights)))

i2 = max(0.0, (q - df) / q * 100) if q > 0 else 0.0

return {

"estimate": pooled,

"lower": pooled - 1.96 * se,

"upper": pooled + 1.96 * se,

"tau2": tau2,

"q": q,

"i2": i2,

}

def leave_one_out(rows, function):

output = []

for index, row in enumerate(rows):

subset = rows[:index] + rows[index + 1 :]

result = function(subset)

output.append({"omitted": row[0], **result})

return output

def save_figure(fig: plt.Figure, stem: str) -> None:

fig.savefig(OUT / f"{stem}.svg", format="svg")

fig.savefig(OUT / f"{stem}_300dpi.png", format="png", dpi=300)

plt.close(fig)

def forest_proportions(

rows: list[tuple[str, int, int]],

pooled: dict,

title: str,

xlabel: str,

stem: str,

x_max: float,

footnote: str,

) -> None:

count = len(rows)

y_values = np.arange(count, 0, -1)

fig, ax = plt.subplots(figsize=(9.2, 0.62 * count + 2.2))

for y, (label, events, total) in zip(y_values, rows):

observed = events / total

lower, upper = wilson_ci(events, total)

ax.plot([lower, upper], [y, y], color=BLUE, lw=1.7, zorder=1)

ax.scatter(observed, y, color=BLUE, edgecolor="white", linewidth=0.8, s=48, zorder=2)

ax.text(x_max * 1.02, y, f"{events}/{total} {observed*100:.1f}% ({lower*100:.1f}–{upper*100:.1f})", va="center", fontsize=8)

pooled_y = 0

centre, lower, upper = pooled["estimate"], pooled["lower"], pooled["upper"]

diamond = Polygon(

[(lower, pooled_y), (centre, pooled_y + 0.22), (upper, pooled_y), (centre, pooled_y - 0.22)],

closed=True,

facecolor=ORANGE,

edgecolor=ORANGE,

zorder=3,

)

ax.add_patch(diamond)

ax.text(x_max * 1.02, pooled_y, f"{centre*100:.1f}% ({lower*100:.1f}–{upper*100:.1f})", va="center", fontsize=8, color=NAVY, weight="bold")

ax.set_yticks(list(y_values) + [pooled_y])

ax.set_yticklabels([r[0] for r in rows] + ["Random-effects model"], fontsize=9)

ax.set_xlim(0, x_max)

ax.set_ylim(-0.8, count + 0.8)

ax.set_xlabel(xlabel)

ax.set_title(title, loc="left", weight="bold", color=NAVY, pad=12)

ax.grid(axis="x", color="#DDE3E8", lw=0.8)

ax.spines[["top", "right", "left"]].set_visible(False)

ax.tick_params(axis="y", length=0)

fig.text(0.12, 0.015, footnote, ha="left", va="bottom", fontsize=7.5, color=GREY)

fig.subplots_adjust(left=0.22, right=0.73, bottom=0.18, top=0.88)

save_figure(fig, stem)

def forest_fev1() -> None:

pooled = dl_mean_difference(FEV1)

count = len(FEV1)

y_values = np.arange(count, 0, -1)

fig, ax = plt.subplots(figsize=(9.2, 4.8))

for y, (label, estimate, se) in zip(y_values, FEV1):

lower, upper = estimate - 1.96 * se, estimate + 1.96 * se

ax.plot([lower, upper], [y, y], color=BLUE, lw=1.7)

ax.scatter(estimate, y, color=BLUE, edgecolor="white", linewidth=0.8, s=48, zorder=2)

ax.text(0.47, y, f"{estimate:+.2f} L ({lower:+.2f} to {upper:+.2f})", va="center", fontsize=8)

centre, lower, upper = pooled["estimate"], pooled["lower"], pooled["upper"]

diamond = Polygon(

[(lower, 0), (centre, 0.22), (upper, 0), (centre, -0.22)],

closed=True,

facecolor=ORANGE,

edgecolor=ORANGE,

)

ax.add_patch(diamond)

ax.text(0.47, 0, f"{centre:+.2f} L ({lower:+.2f} to {upper:+.2f})", va="center", fontsize=8, color=NAVY, weight="bold")

ax.axvline(0, color=GREY, lw=1, ls="--")

ax.set_yticks(list(y_values) + [0])

ax.set_yticklabels([r[0] for r in FEV1] + ["Random-effects model"], fontsize=9)

ax.set_xlim(-0.10, 0.46)

ax.set_ylim(-0.8, count + 0.8)

ax.set_xlabel("Mean change in FEV₁ (L); positive values favour postoperative improvement")

ax.set_title("Exploratory change in FEV₁ after combined resection and volume reduction", loc="left", weight="bold", color=NAVY, pad=12)

ax.grid(axis="x", color="#DDE3E8", lw=0.8)

ax.spines[["top", "right", "left"]].set_visible(False)

ax.tick_params(axis="y", length=0)

fig.text(

0.12,

0.02,

"*Mixed pulmonary-nodule/neoplasm cohort. SD of paired change was imputed in three studies; primary assumption r=0.50.",

fontsize=7.5,

color=GREY,

)

fig.subplots_adjust(left=0.22, right=0.76, bottom=0.20, top=0.86)

save_figure(fig, "Figure_4_FEV1_change")

def rounded_box(ax, xy, width, height, text, *, fc="white", ec=BLUE, fontsize=8, weight="normal", radius=0.02):

x, y = xy

box = FancyBboxPatch(

(x, y), width, height,

boxstyle=f"round,pad=0.012,rounding_size={radius}",

transform=ax.transAxes,

facecolor=fc,

edgecolor=ec,

linewidth=1.2,

)

ax.add_patch(box)

ax.text(x + width / 2, y + height / 2, text, transform=ax.transAxes, ha="center", va="center", fontsize=fontsize, weight=weight, color=NAVY, wrap=True)

return box

def arrow(ax, start, end, color=NAVY, lw=1.3):

ax.add_patch(FancyArrowPatch(start, end, transform=ax.transAxes, arrowstyle="-|>", mutation_scale=12, lw=lw, color=color))

def prisma_figure() -> None:

fig, ax = plt.subplots(figsize=(8.5, 10.5))

ax.set_axis_off()

ax.text(0.5, 0.965, "Identification of studies via databases and registers", transform=ax.transAxes, ha="center", va="center", fontsize=14, weight="bold", color=NAVY)

rounded_box(ax, (0.08, 0.78), 0.34, 0.12, "Records identified\nDatabases and registers (n = 1,007)", fc=LIGHT, ec=BLUE, fontsize=10, weight="bold")

rounded_box(ax, (0.58, 0.75), 0.34, 0.18, "Records removed before screening\nDuplicates (n = 0)\nAutomation exclusions (n = 0)\nOther reasons (n = 0)", fc="#F7F8FA", ec=GREY, fontsize=9)

rounded_box(ax, (0.08, 0.60), 0.34, 0.10, "Records screened\n(n = 1,007)", fc=LIGHT, ec=BLUE, fontsize=10, weight="bold")

rounded_box(ax, (0.58, 0.60), 0.34, 0.10, "Records excluded\n(n = 985)", fc="#F7F8FA", ec=GREY, fontsize=10)

rounded_box(ax, (0.08, 0.43), 0.34, 0.10, "Reports sought for retrieval\n(n = 22)", fc=LIGHT, ec=BLUE, fontsize=10, weight="bold")

rounded_box(ax, (0.58, 0.43), 0.34, 0.10, "Reports not retrieved\n(n = 2)", fc="#F7F8FA", ec=GREY, fontsize=10)

rounded_box(ax, (0.08, 0.26), 0.34, 0.10, "Reports assessed for eligibility\n(n = 20)", fc=LIGHT, ec=BLUE, fontsize=10, weight="bold")

rounded_box(ax, (0.58, 0.22), 0.34, 0.18, "Reports excluded (n = 10)\nIneligible / insufficient data (n = 6)\nSuperseded / overlapping (n = 4)", fc="#F7F8FA", ec=GREY, fontsize=9)

rounded_box(ax, (0.27, 0.05), 0.46, 0.11, "Unique datasets included in qualitative synthesis (n = 10)\nDatasets contributing to ≥1 quantitative synthesis (n = 7)", fc="#E4F3EE", ec=TEAL, fontsize=10, weight="bold")

arrow(ax, (0.42, 0.84), (0.58, 0.84))

arrow(ax, (0.25, 0.78), (0.25, 0.70))

arrow(ax, (0.42, 0.65), (0.58, 0.65))

arrow(ax, (0.25, 0.60), (0.25, 0.53))

arrow(ax, (0.42, 0.48), (0.58, 0.48))

arrow(ax, (0.25, 0.43), (0.25, 0.36))

arrow(ax, (0.42, 0.31), (0.58, 0.31))

arrow(ax, (0.25, 0.26), (0.42, 0.16))

fig.text(0.5, 0.015, "PRISMA 2020 flow diagram. Counts refer to reports unless labelled as unique datasets.", ha="center", fontsize=8, color=GREY)

save_figure(fig, "Figure_1_PRISMA")

def draw_lungs(ax, centre=(0.16, 0.66), scale=1.0, target=True, resected=False):

cx, cy = centre

\# Patient's right lung is displayed on the viewer's left, following

\# standard coronal radiological orientation.

ax.add_patch(Ellipse((cx - 0.075 * scale, cy), 0.14 * scale, 0.30 * scale, transform=ax.transAxes, facecolor="#F8C8C8", edgecolor=NAVY, lw=1.2))

ax.add_patch(Ellipse((cx + 0.075 * scale, cy), 0.14 * scale, 0.30 * scale, transform=ax.transAxes, facecolor="#E8EEF2", edgecolor=NAVY, lw=1.2))

ax.add_patch(FancyBboxPatch((cx - 0.018 * scale, cy - 0.13 * scale), 0.036 * scale, 0.25 * scale, boxstyle="round,pad=0.002", transform=ax.transAxes, facecolor="white", edgecolor="none"))

ax.plot([cx, cx], [cy + 0.19 * scale, cy + 0.07 * scale], transform=ax.transAxes, color=TEAL, lw=5, solid_capstyle="round")

ax.plot([cx, cx - 0.055 * scale], [cy + 0.07 * scale, cy], transform=ax.transAxes, color=TEAL, lw=3)

ax.plot([cx, cx + 0.055 * scale], [cy + 0.07 * scale, cy], transform=ax.transAxes, color=TEAL, lw=3)

tx, ty = cx - 0.085 * scale, cy + 0.085 * scale

if target and not resected:

ax.add_patch(Circle((tx, ty), 0.027 * scale, transform=ax.transAxes, facecolor=ORANGE, edgecolor="#8A3B0A", lw=1.0))

ax.add_patch(Circle((tx, ty), 0.060 * scale, transform=ax.transAxes, fill=False, edgecolor=RED, lw=1.3, ls="--"))

if resected:

ax.add_patch(Polygon([(cx - 0.142 * scale, cy + 0.13 * scale), (cx - 0.012 * scale, cy + 0.13 * scale), (cx - 0.055 * scale, cy + 0.015 * scale)], transform=ax.transAxes, facecolor="white", edgecolor=RED, lw=1.4, ls="--"))

ax.text(cx - 0.16 * scale, cy + 0.18 * scale, "R", transform=ax.transAxes, fontsize=8, weight="bold", color=NAVY)

def physiological_schematic() -> None:

fig, ax = plt.subplots(figsize=(14, 8))

ax.set_axis_off()

ax.text(0.5, 0.965, "Mechanistic rationale for combined lung cancer resection and volume reduction", transform=ax.transAxes, ha="center", va="center", fontsize=16, weight="bold", color=NAVY)

ax.text(0.5, 0.927, "Illustrative schematic—not a patient image or data-derived anatomical reconstruction", transform=ax.transAxes, ha="center", va="center", fontsize=9, color=GREY)

\# Column headers

rounded_box(ax, (0.03, 0.82), 0.27, 0.07, "", fc=NAVY, ec=NAVY, fontsize=11, weight="bold")

rounded_box(ax, (0.365, 0.82), 0.27, 0.07, "", fc=TEAL, ec=TEAL, fontsize=11, weight="bold")

rounded_box(ax, (0.70, 0.82), 0.27, 0.07, "", fc="#4B3F72", ec="#4B3F72", fontsize=11, weight="bold")

\# White text overlays on dark headers

for x, label in [(0.165, "A Selection phenotype"), (0.50, "B Surgical strategy"), (0.835, "C Mechanism and outcomes")]:

ax.text(x, 0.855, label, transform=ax.transAxes, color="white", ha="center", va="center", fontsize=11, weight="bold")

\# A: phenotype

draw_lungs(ax, centre=(0.165, 0.61), scale=1.12, target=True)

ax.text(0.165, 0.405, "Right-upper-lobe tumour within a\npoorly perfused emphysematous target", transform=ax.transAxes, ha="center", fontsize=9, weight="bold", color=NAVY)

rounded_box(ax, (0.055, 0.20), 0.22, 0.16, "CT: heterogeneous destruction + hyperinflation\nPerfusion: low target-lobe contribution\nPhysiology: reduced FEV₁ / DLCO, increased RV\nOncology: resectable disease", fc=LIGHT, ec=BLUE, fontsize=8)

\# B: aligned resection target

draw_lungs(ax, centre=(0.50, 0.61), scale=1.12, target=False, resected=True)

ax.text(0.50, 0.405, "Resection target remains anatomically aligned\nwith the right-upper-lobe tumour/defect", transform=ax.transAxes, ha="center", fontsize=9, weight="bold", color=NAVY)

rounded_box(ax, (0.385, 0.20), 0.23, 0.16, "Eligible operative concepts\n1. Formal LVRS + cancer resection\n2. LVRS-like sublobar target resection\n3. Lobectomy of a poorly functioning target lobe\nPreserve well-perfused parenchyma", fc="#E4F3EE", ec=TEAL, fontsize=8)

\# C: mechanism and balanced outcomes

rounded_box(ax, (0.72, 0.64), 0.23, 0.13, "↓ Residual volume and hyperinflation\n↑ Diaphragmatic curvature\n↑ Elastic recoil and expiratory flow\nPotentially improved V/Q matching", fc="#F2EFF8", ec="#6C5A94", fontsize=8.5)

arrow(ax, (0.835, 0.64), (0.835, 0.56), color="#6C5A94")

rounded_box(ax, (0.72, 0.43), 0.23, 0.12, "Potential benefit\nPreserved or improved FEV₁, dyspnoea,\nexercise capacity and quality of life", fc="#E4F3EE", ec=GREEN, fontsize=8.5)

rounded_box(ax, (0.72, 0.20), 0.23, 0.16, "Observed evidence (very low certainty)\nMortality: 3/113 (2.7%) observed;\n7.3% model estimate (95% CI 3.4–15.1)\nProlonged air leak: 38.0%\nFEV₁: +0.26 L (exploratory)", fc=PALE_ORANGE, ec=ORANGE, fontsize=8.2)

arrow(ax, (0.30, 0.56), (0.365, 0.56), color=TEAL, lw=2)

arrow(ax, (0.635, 0.56), (0.70, 0.56), color=TEAL, lw=2)

fig.text(0.5, 0.035, "Anatomical suitability does not establish oncologic equivalence or superiority over non-operative therapy; multidisciplinary selection remains essential.", ha="center", fontsize=9, color=GREY)

save_figure(fig, "Figure_6_mechanistic_schematic")

def write_results() -> None:

mortality = dl_logit_proportion(MORTALITY)

pal = dl_logit_proportion(PAL)

recurrence = dl_logit_proportion(RECURRENCE)

fev1 = dl_mean_difference(FEV1)

strict_mortality = dl_logit_proportion([row for row in MORTALITY if not row[0].endswith("*")])

strict_pal = dl_logit_proportion([row for row in PAL if not row[0].endswith("*")])

results = {

"software": {

"python": "3.12.13",

"numpy": "2.3.5",

"scipy": "1.17.0",

"matplotlib": "3.10.8",

},

"mortality": mortality,

"mortality_leave_one_out": leave_one_out(MORTALITY, dl_logit_proportion),

"mortality_strict_confirmed_malignancy": strict_mortality,

"pal": pal,

"pal_leave_one_out": leave_one_out(PAL, dl_logit_proportion),

"pal_strict_confirmed_malignancy": strict_pal,

"fev1": fev1,

"fev1_leave_one_out": leave_one_out(FEV1, dl_mean_difference),

"recurrence": recurrence,

}

(OUT / "analysis_results.json").write_text(json.dumps(results, indent=2), encoding="utf-8")

def main() -> None:

write_results()

prisma_figure()

mortality = dl_logit_proportion(MORTALITY)

forest_proportions(

MORTALITY,

mortality,

"Perioperative mortality after combined resection and volume reduction",

"Observed proportion",

"Figure_2_perioperative_mortality",

0.52,

"Markers show observed study proportions with Wilson 95% CIs. The pooled diamond is the random-effects logit estimate with 0.5 continuity correction. *Mixed cohort.",

)

forest_proportions(

PAL,

dl_logit_proportion(PAL),

"Prolonged air leak after combined resection and volume reduction",

"Observed proportion",

"Figure_3_prolonged_air_leak",

0.83,

"Markers show observed study proportions with Wilson 95% CIs. Definitions varied (>7, ≥7, >14 days, or non-standardised reporting). *Mixed cohort.",

)

forest_fev1()

forest_proportions(

RECURRENCE,

dl_logit_proportion(RECURRENCE),

"Reported cancer recurrence after combined resection and volume reduction",

"Observed proportion",

"Figure_5_reported_recurrence",

0.72,

"Markers show observed proportions with Wilson 95% CIs. Follow-up, staging, nodal assessment and recurrence definitions were heterogeneous; the pooled estimate is exploratory.",

)

physiological_schematic()

if __name__ == "__main__":

main()
