# Supplementary material for: Outcomes of combined lung cancer resection and lung volume reduction surgery in adults with severe emphysema; a systematic review and meta-analysis
Source: Front Oncol. 2026 Aug 19;16:1916431. doi: 10.3389/fonc.2026.1916431 (PMC13533703; doi:10.3389/fonc.2026.1916431)
Supplement: Supplementary file 6 [file Table5.docx]

{

"software": {

"python": "3.12.13",

"numpy": "2.3.5",

"scipy": "1.17.0",

"matplotlib": "3.10.8"

},

"mortality": {

"events": 3,

"participants": 113,

"crude": 0.02654867256637168,

"estimate": 0.07326184970637611,

"lower": 0.03398222329530527,

"upper": 0.15085416005631866,

"tau2": 0.0,

"q": 4.476440305184254,

"i2": 0.0

},

"mortality_leave_one_out": [

{

"omitted": "Choong 2004",

"events": 3,

"participants": 92,

"crude": 0.03260869565217391,

"estimate": 0.08118840387554399,

"lower": 0.03654240446759976,

"upper": 0.17071587485953443,

"tau2": 0.0,

"q": 3.6783349517646062,

"i2": 0.0

},

{

"omitted": "McKenna 1996",

"events": 3,

"participants": 102,

"crude": 0.029411764705882353,

"estimate": 0.07696016150933431,

"lower": 0.03457670979562782,

"upper": 0.16254908145339053,

"tau2": 0.0,

"q": 4.289912930197676,

"i2": 0.0

},

{

"omitted": "Caviezel 2018",

"events": 3,

"participants": 99,

"crude": 0.030303030303030304,

"estimate": 0.0784902022755405,

"lower": 0.035285202477132085,

"upper": 0.16552087885379976,

"tau2": 0.0,

"q": 4.113829359626124,

"i2": 0.0

},

{

"omitted": "DeRose 1998*",

"events": 2,

"participants": 99,

"crude": 0.020202020202020204,

"estimate": 0.06663655866887189,

"lower": 0.02758431465864734,

"upper": 0.15231652505130816,

"tau2": 0.0,

"q": 4.273174208956552,

"i2": 0.0

},

{

"omitted": "Edwards 2001",

"events": 1,

"participants": 99,

"crude": 0.010101010101010102,

"estimate": 0.04522863989534491,

"lower": 0.016983417227170446,

"upper": 0.11495536120691488,

"tau2": 0.0,

"q": 1.6912740542780718,

"i2": 0.0

},

{

"omitted": "Pompeo 2003",

"events": 3,

"participants": 97,

"crude": 0.030927835051546393,

"estimate": 0.07936242368602375,

"lower": 0.03569059930601073,

"upper": 0.1672062376482631,

"tau2": 0.0,

"q": 3.989946102420345,

"i2": 0.0

},

{

"omitted": "Tang 2009*",

"events": 3,

"participants": 90,

"crude": 0.03333333333333333,

"estimate": 0.08181310302526021,

"lower": 0.036834704783618874,

"upper": 0.17191117358053012,

"tau2": 0.0,

"q": 3.5561385550447637,

"i2": 0.0

}

],

"mortality_strict_confirmed_malignancy": {

"events": 2,

"participants": 76,

"crude": 0.02631578947368421,

"estimate": 0.07641409407415224,

"lower": 0.03018721584212694,

"upper": 0.1802715512616,

"tau2": 0.0,

"q": 3.468267379215196,

"i2": 0.0

},

"pal": {

"events": 28,

"participants": 76,

"crude": 0.3684210526315789,

"estimate": 0.3804464986907184,

"lower": 0.26916954137391513,

"upper": 0.5058829924695405,

"tau2": 0.05586035003582353,

"q": 4.779496263735933,

"i2": 16.30917194454783

},

"pal_leave_one_out": [

{

"omitted": "Choong 2004",

"events": 17,

"participants": 55,

"crude": 0.3090909090909091,

"estimate": 0.32904231262682904,

"lower": 0.219255525747278,

"upper": 0.4613200980951253,

"tau2": 0.0,

"q": 2.325655994068235,

"i2": 0.0

},

{

"omitted": "McKenna 1996",

"events": 23,

"participants": 65,

"crude": 0.35384615384615387,

"estimate": 0.35832207430726537,

"lower": 0.22927340662685794,

"upper": 0.5117753082193739,

"tau2": 0.13538987322703142,

"q": 4.461095470533395,

"i2": 32.75194355700927

},

{

"omitted": "Caviezel 2018",

"events": 25,

"participants": 62,

"crude": 0.4032258064516129,

"estimate": 0.4154375805556854,

"lower": 0.2981327966757419,

"upper": 0.5431778922237065,

"tau2": 0.011166133254976666,

"q": 3.1234605110568534,

"i2": 3.952683589877669

},

{

"omitted": "DeRose 1998*",

"events": 25,

"participants": 62,

"crude": 0.4032258064516129,

"estimate": 0.4154375805556854,

"lower": 0.2981327966757419,

"upper": 0.5431778922237065,

"tau2": 0.011166133254976666,

"q": 3.1234605110568534,

"i2": 3.952683589877669

},

{

"omitted": "Pompeo 2003",

"events": 22,

"participants": 60,

"crude": 0.36666666666666664,

"estimate": 0.37148000314226387,

"lower": 0.23073301417217926,

"upper": 0.5380341883772556,

"tau2": 0.17830153704290144,

"q": 4.778462431592159,

"i2": 37.21829892046644

}

],

"pal_strict_confirmed_malignancy": {

"events": 25,

"participants": 62,

"crude": 0.4032258064516129,

"estimate": 0.4154375805556854,

"lower": 0.2981327966757419,

"upper": 0.5431778922237065,

"tau2": 0.011166133254976666,

"q": 3.1234605110568534,

"i2": 3.952683589877669

},

"fev1": {

"estimate": 0.257509856459321,

"lower": 0.1956668205766563,

"upper": 0.3193528923419857,

"tau2": 0.0,

"q": 2.341111914713651,

"i2": 0.0

},

"fev1_leave_one_out": [

{

"omitted": "Choong 2004",

"estimate": 0.23941495935328852,

"lower": 0.16556854654775638,

"upper": 0.31326137215882066,

"tau2": 0.0,

"q": 1.5688317921573611,

"i2": 0.0

},

{

"omitted": "DeRose 1998*",

"estimate": 0.26004751044149765,

"lower": 0.19302777474549201,

"upper": 0.32706724613750326,

"tau2": 0.0003128257422617904,

"q": 2.1923724283750254,

"i2": 8.774623594295557

},

{

"omitted": "Pompeo 2003",

"estimate": 0.24259586419624637,

"lower": 0.16273451618793638,

"upper": 0.32245721220455636,

"tau2": 1.3931065161253672e-05,

"q": 2.0049016879620165,

"i2": 0.24448520301257656

},

{

"omitted": "Tang 2009*",

"estimate": 0.28219372225205486,

"lower": 0.21118291599194972,

"upper": 0.35320452851216,

"tau2": 0.0,

"q": 0.4193452764527924,

"i2": 0.0

}

],

"recurrence": {

"events": 12,

"participants": 58,

"crude": 0.20689655172413793,

"estimate": 0.2514882820958302,

"lower": 0.15427950007895308,

"upper": 0.38226206861989753,

"tau2": 0.0,

"q": 2.277129392073466,

"i2": 0.0

}

}
